# Functional genomics pipeline identifies CRL4 inhibition for the treatment of ovarian cancer

**DOI:** 10.1002/ctm2.70078

**Published:** 2025-01-24

**Authors:** Sally E. Claridge, Shalini Nath, Anneliese Baum, Richard Farias, Julie‐Ann Cavallo, Nile M. Rizvi, Lamberto De Boni, Eric Park, Genesis Lara Granados, Matthew Hauesgen, Ruben Fernandez‐Rodriguez, Eda Nur Kozan, Evgeny Kanshin, Khoi Q. Huynh, Peng‐Jen Chen, Kenneth Wu, Beatrix Ueberheide, Juan Miguel Mosquera, Fred R. Hirsch, Robert J. DeVita, Olivier Elemento, Chantal Pauli, Zhen‐Qiang Pan, Benjamin D. Hopkins

**Affiliations:** ^1^ Department of Physiology and Biophysics Weill Cornell Medicine New York New York USA; ^2^ Englander Institute for Precision Medicine, Weill Cornell Medicine, New York Presbyterian Hospital New York New York USA; ^3^ Department of Genetics and Genomic Sciences Icahn School of Medicine at Mount Sinai New York New York USA; ^4^ Department of Oncological Sciences Icahn School of Medicine at Mount Sinai New York New York USA; ^5^ Tisch Cancer Institute, Icahn School of Medicine at Mount Sinai New York New York USA; ^6^ Department of Pathology and Laboratory Medicine Weill Cornell Medicine New York New York USA; ^7^ Department of Biochemistry and Molecular Pharmacology New York University School of Medicine New York New York USA; ^8^ Proteomics Laboratory New York University School of Medicine New York New York USA; ^9^ Department of Pharmacological Sciences Icahn School of Medicine at Mount Sinai New York New York USA; ^10^ Drug Discovery Institute, Icahn School of Medicine at Mount Sinai New York New York USA; ^11^ Department of Neurology New York University Grossman School of Medicine New York New York USA; ^12^ Department of Medicine, Hematology, and Medical Oncology Icahn School of Medicine at Mount Sinai New York New York USA; ^13^ Department of Pathology, Molecular and Cell‐Based Medicine Icahn School of Medicine at Mount Sinai New York New York USA; ^14^ Institute for Computational Biomedicine, Weill Cornell Medicine New York New York USA; ^15^ Clinical and Translational Science Center, Weill Cornell Medicine New York New York USA; ^16^ Department of Pathology and Molecular Pathology University Hospital Zurich Zurich Switzerland

**Keywords:** CRL4, functional genomics, ovarian cancer, precision oncology

## Abstract

**Background:**

The goal of precision oncology is to find effective therapeutics for every patient. Through the inclusion of emerging therapeutics in a high‐throughput drug screening platform, our functional genomics pipeline inverts the common paradigm to identify patient populations that are likely to benefit from novel therapeutic strategies.

**Approach:**

Utilizing drug screening data across a panel of 46 cancer cell lines from 11 tumor lineages, we identified an ovarian cancer‐specific sensitivity to the first‐in‐class CRL4 inhibitors KH‐4‐43 and 33‐11. CRL4 (i.e., Cullin‐4 RING E3 ubiquitin ligase) is known to be dysregulated in a variety of cancer contexts, making it an attractive therapeutic target. Unlike proteasome inhibitors that are associated with broad toxicity, CRL4 inhibition offers the potential for tumor‐specific effects.

**Results:**

We observed that CRL4 inhibition negatively regulates core gene signatures that are upregulated in ovarian tumors and significantly slowed tumor growth as compared to the standard of care, cisplatin, in OVCAR8 xenografts. Building on this, we performed combination drug screening in conjunction with proteomic and transcriptomic profiling to identify ways to improve the antitumor effects of CRL4 inhibition in ovarian cancer models. CRL4 inhibition consistently resulted in activation of the mitogen‐activated protein kinase (MAPK) signaling cascade at both the transcriptomic and protein levels, suggesting that survival signaling is induced in response to CRL4 inhibition. These observations were concordant with the results of the combination drug screens in seven ovarian cancer cell lines that showed CRL4 inhibition cooperates with MEK inhibition. Preclinical studies in OVCAR8 and A2780 xenografts confirmed the therapeutic potential of the combination of KH‐4‐43 and trametinib, which extended overall survival and slowed tumor progression relative to either single agent or the standard of care.

**Conclusions:**

Together, these data demonstrate the prospective utility of functional modeling pipelines for therapeutic development and underscore the clinical potential of CRL4 inhibition in the ovarian cancer context.

**Highlights:**

- A precision medicine pipeline identifies ovarian cancer sensitivity to CRL4 inhibitors.
- CRL4 inhibition induces activation of MAPK signalling as identified by RNA sequencing, proteomics, and phosphoproteomics.
- Inhibitor combinations that target both CRL4 and this CRL4 inhibitor‐induced survival signalling enhance ovarian cancer sensitivity to treatment.

## Graphical abstract

- A precision medicine pipeline identifies ovarian cancer sensitivity to CRL4 inhibitors.
- CRL4 inhibition induces activation of mitogen‐activated protein kinase signalling as identified by RNA sequencing, proteomics, and phosphoproteomics.
- Inhibitor combinations that target both CRL4 and this CRL4 inhibitor‐induced survival signalling enhance ovarian cancer sensitivity to treatment.

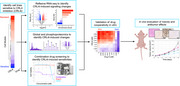

## 1 INTRODUCTION

The dominant paradigm of precision oncology is to target tumour‐specific vulnerabilities to improve patient outcomes while minimizing toxicity. The first wave of precision oncology targets were the protein products of driver mutations like *BRAF^V600E^* in melanoma, *EGFR* mutations in lung cancer, or *HER2* amplifications in breast cancer.^1^, ^2^ While the agents targeting these driver mutations have demonstrated significant benefits, their clinical impact frequently leaves room for improvement since many tumours demonstrate primary and/or acquired resistance, thereby limiting the efficacy of these agents. To circumvent the shortcomings of these drugs, we have developed a functional genomics pipeline that seeks to identify tumour‐specific drug sensitivities to enhance therapeutic efficacy with the goal of inducing long‐term responses. By taking a nonbiased functional genomics approach, this pipeline can interrogate which patient populations may benefit from specific drugs and identify combinations that cooperate with these agents. Including novel agents in our high‐throughput drug screening panel allows the accumulation of sensitivity data in a wide array of tumour lineages and genomic contexts and provides insight into their antitumor capacities while highlighting features that drive tumour‐specific dependencies. We employed this drug‐centric precision oncology paradigm to evaluate the therapeutic capacity of KH‐4‐43 and 33‐11, two first‐in‐class small molecule inhibitors of the cullin 4 (CUL4) RING (really interesting new gene) E3 ubiquitin (Ub) ligase (CRL4).^3^

The Ub‐proteasome system (UPS) is essential for regulating protein homeostasis in healthy cells and is one of the primary mechanisms by which intracellular proteins are degraded.^4^, ^5^ Coordinated actions by E1 (Ub‐activating), E2 (Ub‐conjugating), and E3 (Ub‐ligating) enzymes result in covalent linkage of Ub moieties to target proteins, tagging them for degradation by the 26S proteasome.^4^, ^6^ By modulating levels of regulatory proteins, Ub‐mediated degradation plays a key role in many important cellular processes and pathways, for example, cell cycle progression, immune response, and apoptosis.^7^, ^8^, ^9^, ^10^ Disruption of this regulatory balance has been associated with numerous health complications, for example, cancer,^10^ diabetes,^11^ neurodegenerative diseases,^12^ and viral infections.^13^ The 26S proteasome inhibitors bortezomib, carfilzomib, and marizomib have shown clinical utility in multiple myeloma and other hematologic cancers.^14^, ^15^, ^16^, ^17^, ^18^, ^19^, ^20^, ^21^ Despite this efficacy, these inhibitors exert broad cytotoxicity through the unbiased inhibition of UPS function^22^ and, as a consequence, have not shown significant utility in the treatment of solid tumours.^23^, ^24^ In order to target the cellular requirements for UPS activity while avoiding broad toxicity of proteasome inhibition, researchers have begun to develop agents that target individual components of the UPS, for example, specific E1, E2, and E3 enzymes, focusing on those that are dysregulated in cancer.^25^, ^26^, ^27^, ^28^ Many E3 ligase components have been observed to have differing expression and/or activity across tissues and cancer types,^29^, ^30^ suggesting that there may be unique requirements for specific E3 ligase activity in various tumour lineages. This underscores the potential for developing targeted interventions that exploit these cell type‐specific dependencies.

First described over two decades ago,^31^, ^32^ CRLs are a distinct sub‐family of RING E3 ligases that utilize one of nine cullin (CUL) scaffolds to bring together a RING protein (e.g., ROC1/RBX1), an adaptor protein (e.g., DDB1), and one of many substrate recognition proteins that work together as a complex to tag substrates for degradation.^33^, ^34^ Multiple CRL complexes have been explicitly linked to chemoresistance mechanisms in ovarian cancer,^35^, ^36^, ^37^ and perturbation of these complexes has shown promise as a potential therapeutic strategy.^25^, ^27^, ^38^ CRLs comprise the largest and most diverse sub‐family of all E3 Ub ligases,^28^, ^39^ but to date, only two types of CRL‐targeting drugs have been approved by the U.S. Food and Drug Administration (FDA) for the treatment of haematological malignancies: the immunomodulatory imide drugs (IMiDs), which include thalidomide and its analogues,^40^, ^41^, ^42^ and pevonedistat (TAK‐924/MLN4924), a NEDD8 (neural precursor cell‐expressed developmentally down‐regulated 8) activating enzyme inhibitor that prevents the activation of nearly all CUL‐based E3 complexes.^43^, ^44^, ^45^

The CUL4‐based subgroup of CRLs is unique in that it includes two scaffolds, CUL4A and CUL4B, with distinct but related structures and functions. The CUL4A/B scaffolds share 80% sequence identity and have highly similar tertiary structures.^46^, ^47^ They are distinct in that CUL4B has an N‐terminal nuclear localization sequence and is thought to predominantly act in the nucleus, while CUL4A is typically found in the cytosol.^48^ In healthy cells, CRL4A and CRL4B have been shown to have partially overlapping pleiotropic effects on the levels of key mediators of embryonic development,^49^ metabolism,^50^, ^51^ DNA repair,^52^, ^53^ and cell cycle progression.^48^, ^54^, ^55^, ^56^ In tumours, CRL4 has been shown to be dysregulated mainly due to overexpression of the CUL4A/B scaffold proteins,^57^, ^58^, ^59^ making it an attractive anti‐cancer target.^60^ Increased CUL4A and CUL4B expression has been observed in neoplastic contexts in humans and mice,^46^, ^61^ for example, increased activity of CRL4B has been linked to epigenetic silencing of the tumour suppressor gene *IGFBP3* in vitro and in vivo^62^ and CRL4A hyperactivity has been shown to facilitate chemoresistance in multiple cancer contexts, notably in cisplatin‐resistant ovarian cancers.^37^, ^63^, ^64^ Despite its known activity in oncogenesis and tumour maintenance, CRL4 is less well understood than other CRLs, for example, CRL1/SCF^30^ and CUL2^(VHL)^.^65^ Recently, two first‐in‐class CRL4 inhibitors, KH‐4‐43 and 33‐11, were described.^3^ Both directly bind to the core ligase sub‐complex of CRL4, inhibit its E3 ligase activity in vitro and in cell‐based assays, and exhibit antitumor activity in vitro and in vivo, with KH‐4‐43 binding more strongly to the catalytic core and generally being more efficacious in killing cancer cells.^3^ By including these two novel agents in our drug library, we conducted a targeted yet nonbiased study of a specific axis of CRL biology and its role in cancer progression and maintenance.

Our precision oncology workflow integrates high‐throughput drug screening, transcriptomic and proteomic profiling, and in vivo modeling to identify effective therapeutic combinations,^66^ and we use a bespoke compound library of clinical trial drugs, experimental therapeutics, and select standard‐of‐care agents to functionally screen models representing diverse lineage and genomic contexts. The parallel screening design allows for direct comparison of tumour responses to new and standard‐of‐care therapeutic regimens, thereby expanding our understanding of tumour‐specific sensitivities and informing drug repurposing efforts. This provides for a greater potential of identifying current and emerging therapies that could benefit specific patient subpopulations that may otherwise have been overlooked due to the lack of tumour‐specific granularity in traditional functional screening workflows. Unlike many other existing functional genomics pipelines, our methodology integrates (1) iterative, large‐scale functional combination screens with our compound of interest to identify drug‐induced sensitivities that can be compared with the drugs the patient would have seen in the clinic and (2) reflex RNA‐sequencing and proteomic analysis of single‐agent treated/untreated cells to characterize drug‐induced changes. These multi‐omic profiles can then be conceptually applied to help understand differences in patient sensitivity and filter candidate drug combinations for those that perturb targets that may drive resistance to the primary agent. By inverting the common precision oncology paradigm to focus on emerging therapeutics, we can interrogate the anticancer capacity of perturbing signalling axes in novel ways and evaluate the pathways that are altered in response to these inhibitors. Given the known dysregulation of CRLs and the observed upregulation of *CUL4A* and *CUL4B* in a variety of cancers, we leveraged this inverse precision medicine approach to identify patient populations that might benefit from CRL4 inhibition and to dissect the functional roles of the CRL4 complex in sensitive tumours. This study is the first to be able to evaluate the transcriptomic, proteomic, signalling, and functional consequences of pharmacological inhibition of CRL4, providing a foundation upon which future studies of inhibiting specific E3 ligases can build.

## 2 RESULTS

### 2.1 High‐throughput functional screening of a diverse panel of cancer cell lines reveals an ovarian cancer‐specific sensitivity to KH‐4‐43 and 33‐11

**FIGURE 1:**
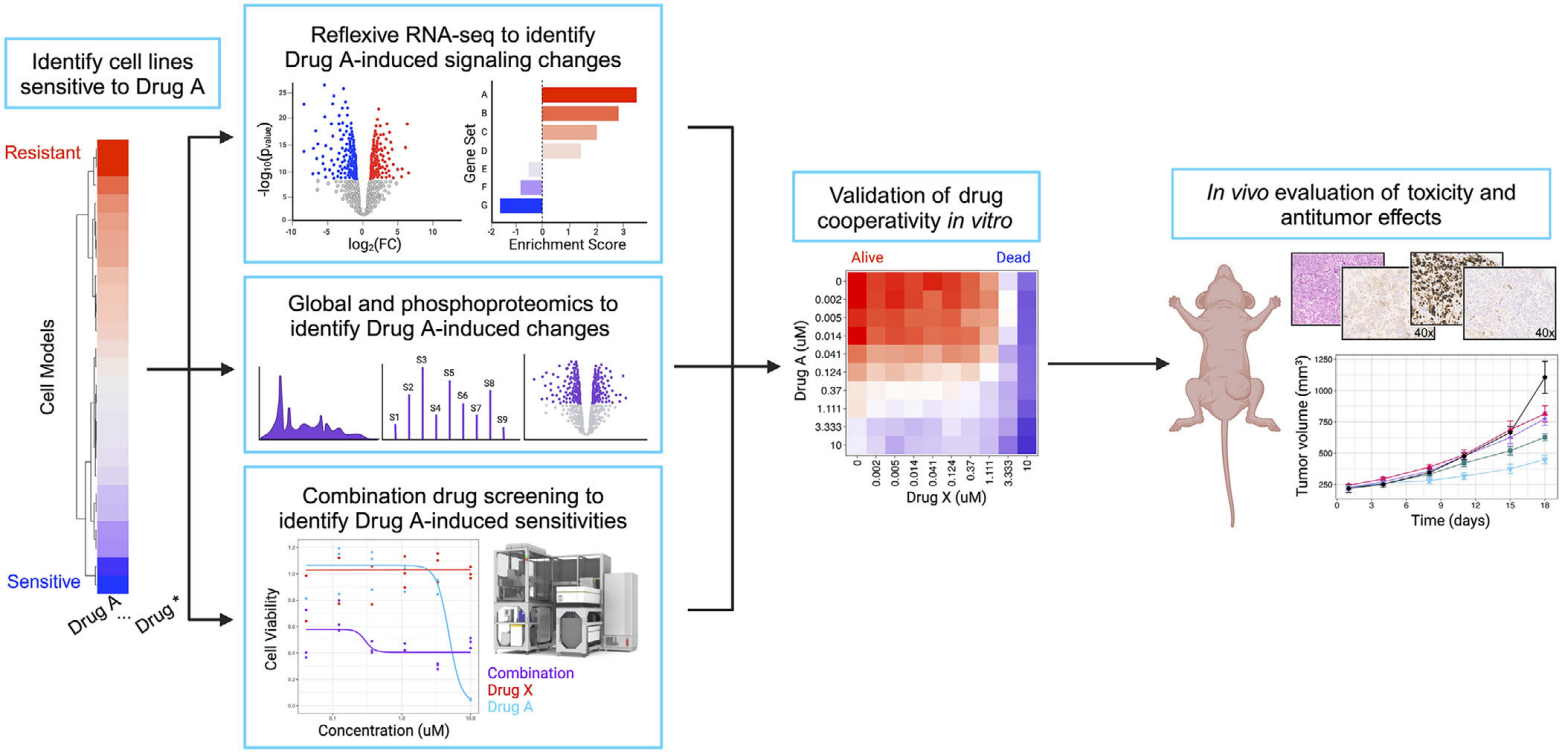
Overview of the functional genomics pipeline. The functional genomics workflow begins with high‐throughput drug screening, followed by combination screening and multi‐omic profiling, and concludes with cooperativity validation in vitro and in vivo (created with BioRender.com).

**FIGURE 2:**
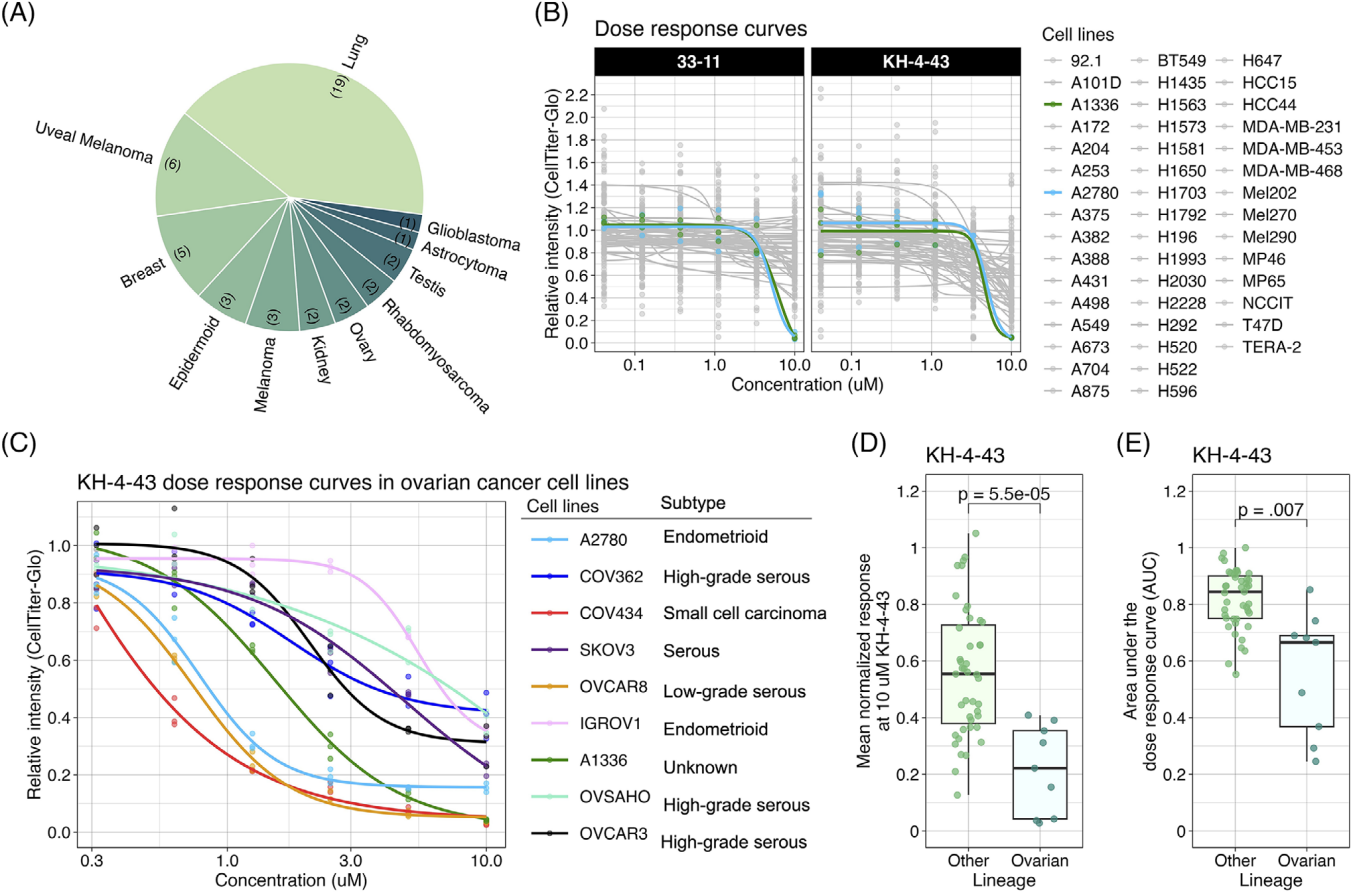
Functional screens of KH‐4‐43 demonstrating the sensitivity of ovarian cancer cell lines to treatment. (A) Distribution of lineages represented in our cancer cell line panel. (B) 33‐11 and KH‐4‐43 dose–response curves across 46 cell lines from 11 lineages screened via our automated system. (C) KH‐4‐43 dose–response curves across our panel of nine ovarian cancer cell lines, including manual validation of dose–response curves in (B). Points represent triplicate measurements; dose–response curves are generated from a four‐parameter logistic model fit to the triplicate data. (D) Comparison of the mean normalized response to 10 µM KH‐4‐43 shows that the nine ovarian cancer lines were significantly more sensitive to the KH‐4‐43 than the 44 cell lines in (B) derived from other lineages (*p* < .0001, *t* [5.44] = 15.891, Welch's two‐sample *t*‐test). (E) The ovarian cell lines also exhibited significantly lower area under the dose‐response curve (AUC) values (*p* = .007, *t* [3.53] = 8.815, Welch's two‐sample *t*‐test). Boxes indicate the 25th to 75th percentile (i.e., the inter‐quartile range), the midlines represent the 50th percentile (i.e., the median), and the whiskers indicate the most extreme values within 1.5 times the inter‐quartile range.

Modern cancer research has benefitted from the pairing of functional screening and multi‐omic assessments. Our functional genomics pipeline provides a standardized workflow through which these data can be integrated at scale, allowing us to build robust compendia of readouts to compare therapeutic strategies and assess the impact of individual compounds of interest (Figure 1). Inverting the paradigm outlined by Dr. Chantal Pauli et al.^66^ that seeks to identify the best therapeutic options for specific patients, we screened 46 cancer cell lines from 11 different lineages to identify tumour models that demonstrated enhanced relative sensitivity to KH‐4‐43 and 33‐11 (Figure 2A,B, Table S1). This approach identified the two ovarian models in our panel, A1336 and A2780, as more sensitive to both CRL4 inhibitors than the other tumour lineages tested (Figure 2B). Supporting our observation of ovarian lineage specificity, CRL4 has been identified as a potential therapeutic target in ovarian cancers,^38^, ^67^ and these screening efforts underscore the promise of developing pharmacological inhibitors of E3 ligase complexes for cancer treatment. To follow up on these observations, we used KH‐4‐43 to validate this sensitivity in an expanded panel of seven additional ovarian cancer cell lines representing various subtypes and housing a diverse array of mutations in known ovarian cancer driver genes such as *TP53*, *PIK3CA*, and *ARID1A* (Figure 2C, Table S2). These ovarian models were significantly more sensitive to KH‐4‐43 treatment compared with the other 44 non‐ovarian cell lines we screened (Figure 2D,E). These results confirm previous studies that showed CRL4 to be dysregulated in ovarian cancer and, being that the ovarian cancer cell lines screened had unique mutational profiles, suggest that the observed sensitivity to CRL4 inhibition may be driven by lineage‐specific dependence rather than by specific genetic aberrations.

### 2.2 KH‐4‐43 exhibits anticancer potential in vivo

**FIGURE 3:**
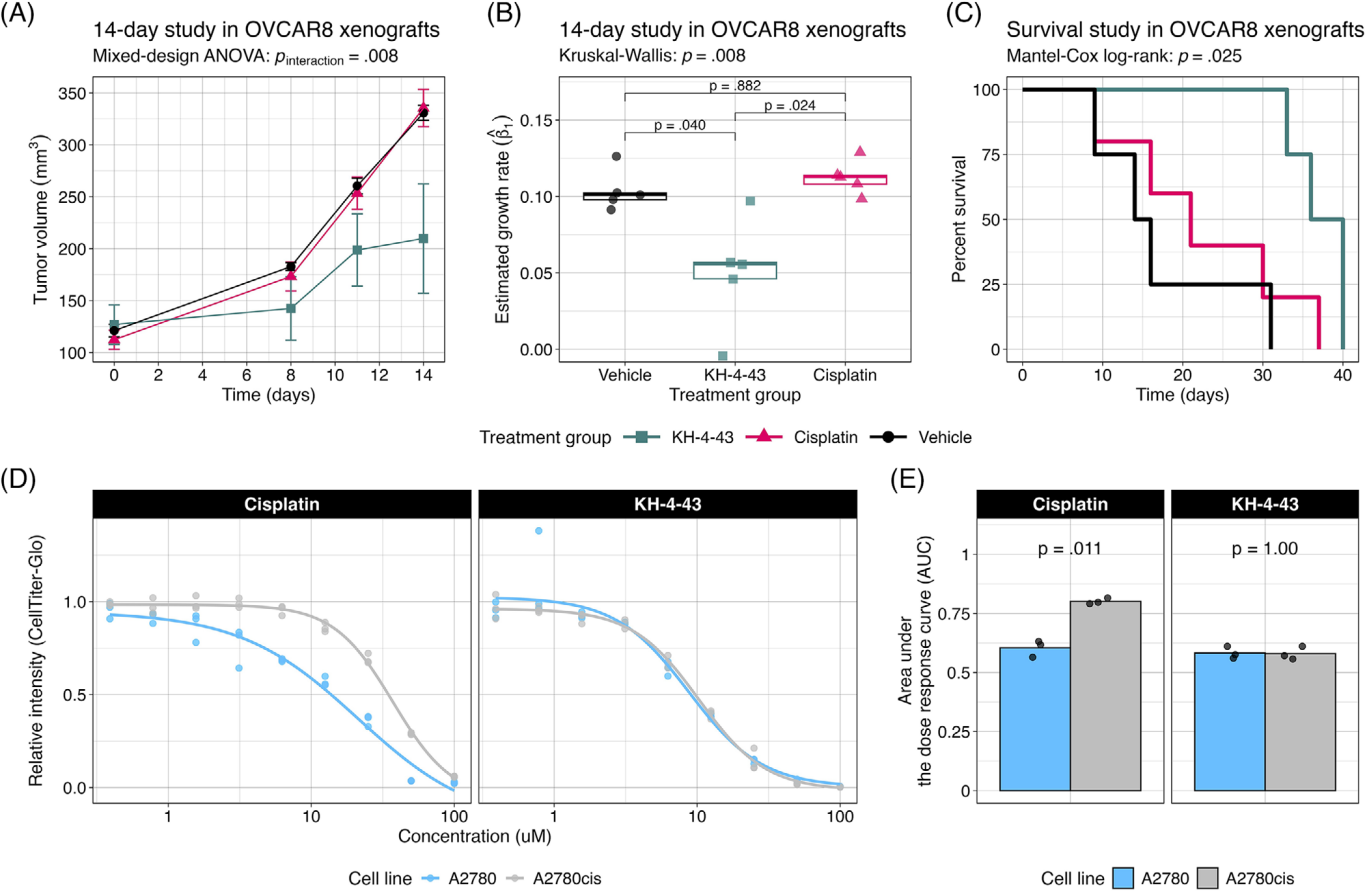
Comparison of antitumor activity between cisplatin and KH‐4‐43 in cisplatin‐resistant ovarian cancer models. We assessed the therapeutic efficacy of KH‐4‐43 compared with cisplatin in OVCAR8 xenografts in female FOXN1^nu^ mice (*n*_group_ = 5). (A) A mixed‐design ANOVA (i.e., a repeated measures two‐way ANOVA model with a between‐subjects factor) was used to assess the therapeutic efficacy of KH‐4‐43 compared with cisplatin in these animals. In a short‐term study, we identified a significant interaction effect of treatment group and time on tumour volume over the 14‐day treatment period (*p*_interaction_ = .008, *F* [2, 12] = 7.528, mixed‐design ANOVA). Data represent the mean and standard error of the mean of the number of animals remaining per treatment arm at each timepoint, which was *n*_group_ = 5 for all groups. (B) Fitting linear models to log_2_‐transformed tumour volumes and estimating tumour growth rates as the slope of the best‐fit line, we see that mean growth rates were significantly different across treatment groups (*p* = .008, χ^2^ = 9.62, df = 2, Kruskal–Wallis test), with KH‐4‐43 eliciting a significantly smaller estimated growth rate as compared with vehicle (*p*_adjusted_ = .0398, *t* [5.05] = −3.10, one‐sided Welch's two‐sample *t*‐test) and cisplatin treatment (*p*_adjusted_ = .024, *t* [4.74] = 3.67, one‐sided Welch's two‐sample *t*‐test). There was no significant difference in median growth rate between the cisplatin‐ and vehicle‐treated animals (*p*_adjusted_ = .882, *t* [7.75] = 1.126, two‐sided Welch's two‐sample *t*‐test). Pairwise *p*‐values are Bonferroni‐corrected for three pairwise tests. Days is indicative of days post‐treatment initiation. Boxes indicate the 25th to 75th percentile (i.e., the inter‐quartile range), the midlines represent the 50th percentile (i.e., the median), and the whiskers indicate the most extreme values within 1.5 times the inter‐quartile range. (C) In a long‐term study, we identified the extension of percent survival in the KH‐4‐43 treatment group (*p* = .025, Mantel‐Cox log‐rank, *c* ^2^ = 7.352, df = 2; *n*_Vehicle_ = 4, *n*_Cisplatin_ = 5, *n*_KH‐4‐43_ = 5). (D) Cisplatin and KH‐4‐43 dose responses in parental A2780 and cisplatin‐resistant A2780cis cells. (E) Comparison of triplicate AUC values for cisplatin and KH‐4‐43 in the A2780 and A2780cis cells (*p*_cisplatin_ = .011, *t* [2.50] = −9.13; *p*_KH‐4‐43_ = 1.00, *t* [4.00] = .120). *p*_cisplatin_ and *p*_KH‐4‐43_ are Bonferroni‐corrected for two pairwise comparisons. Bar height represents the mean AUC across triplicate samples.

Given the reported in vivo antitumor effects of KH‐4‐43 on acute myeloid leukaemia MV4‐11 xenografts,^3^ we evaluated KH‐4‐43 in OVCAR8 xenografts in female FOXN1^nu^ mice to assess its activity against a solid tumour model. OVCAR8 was selected as these cells are resistant to the standard of care, cisplatin,^68^ and we compared the antitumor effects of KH‐4‐43 (50 mg kg^−1^) and cisplatin (3 mg kg^−1^) as single agents. In this manner, we observed that treatment with KH‐4‐43 significantly slowed tumour growth over time (*p*_time_ < .001, *F* [1, 12] = 127.604, mixed‐design ANOVA of time and treatment main effects) (Figure 3A, Figure S1A). Of particular note, there was also a significant interaction effect of treatment and tumour volume over the 14‐day treatment period (*n*_group_ = 5, *p*_interaction_ = .008, *F* [2, 12] = 7.528, mixed‐design ANOVA of time and treatment main effects) (Figure 3A).

To further evaluate the significant interaction of treatment and time, we log_2_‐transformed tumour volumes and estimated the tumour growth rate in each animal by fitting a simple linear model to each subject and extracting the slope parameter, ${\hat{\beta }}_{1}$, which represents the change in log_2_‐transformed tumour volume over time^69^ (Figure S1B). We observed that the median tumour growth rate in the KH‐4‐43‐treated group was significantly lower than those of both the cisplatin (*p*_adjusted_ = .024, *t* [4.74] = 3.67, one‐sided Welch's two‐sample *t*‐test) and vehicle treatment groups^69^, ^70^, ^71^ (*p*_adjusted_ = .040, *t* [5.05] = −3.10, one‐sided Welch's two‐sample *t*‐test) (Figure 3B). As expected in a cisplatin‐resistant model (i.e., OVCAR8 cells), there was no significant difference in growth rate between the cisplatin and vehicle treatment groups (*p*_adjusted_ = .882, *t* [7.75] = 1.126, two‐sided Welch's two‐sample *t*‐test) (Figure 3B). Additionally, in a separate study assessing long‐term effects of treatment on OVCAR8 xenografts, we observed that there were significant main effects of both time (*p*_time_ < .001, *F* [1, 135.61] = 193.570) and treatment (*p*_treatment_ = .009, *F* [2, 136.10] = 4.862) on tumor volume as well as a significant interaction between these two main effects (*p*_interaction_ = .012, *F* [2, 136.35] = 4.610, type III ANOVA of a mixed‐effects model using Satterthwaite's estimation of degrees of freedom) (Figure S1C). Critically for future clinical potential, KH‐4‐43 treatment nearly doubled the median overall survival of animals relative to both vehicle and cisplatin treatment groups (*p* = .0253, Mantel‐Cox log‐rank, *c* ^2^ = 7.352, df = 2) (Figure 3C). To further evaluate the effectiveness of KH‐4‐43 in the cisplatin‐resistant ovarian cancer context, we tested CRL4 inhibition in A2780cis cells, an established cisplatin‐resistant cell line. A2780cis cells were significantly less sensitive to cisplatin treatment compared with parental A2780 cells (*p* = .0055, *t* [2.5] = −9.1, Welch's two‐sample *t*‐test); however, parental A2780 and A2780cis cells were equally sensitive to KH‐4‐43 treatment (*p* = .91, *t* [4] = .12, Welch's two‐sample *t*‐test) (Figure 3D), highlighting the potential of targeting CRL4 in platinum‐refractory ovarian cancer and supporting the potential utility in both the front line and refractory settings.

### 2.3 KH‐4‐43‐treated cell lines display a near‐complete reversal of an ovarian cancer gene expression signature

**FIGURE 4:**
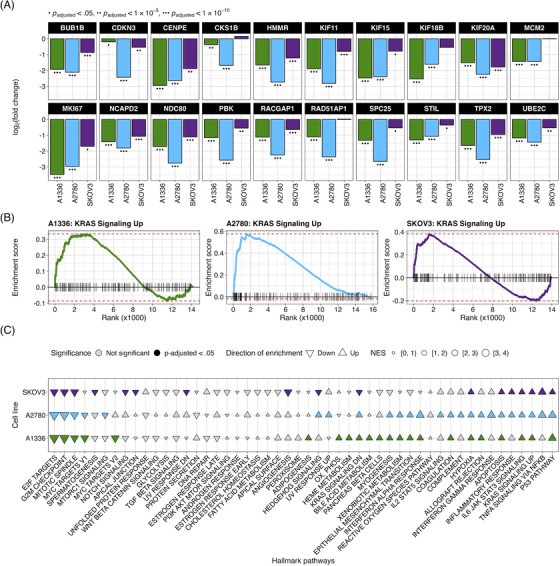
KH‐4‐43 reverses ovarian cancer‐specific gene signature and perturbs proliferative programs in ovarian cancer cells. Three ovarian cancer cell lines were treated with their respective EC30 doses of KH‐4‐43 for 18 h and submitted for transcriptomic profiling (EC30_A2780_ = 3.80 µM, EC30_A1336_ = 3.96 µM, EC30_SKOV3_ = 2.68 µM). Differential gene expression analysis was conducted using the DESeq2 R package (v1.20),^77^ and *p*‐values were computed using Wald tests and were adjusted using the Benjamini–Hochberg method. Genes were considered significantly differentially expressed in treated cells as compared with control if their *p*_adjusted_ < .05 and |log_2_(fold change)| > 1. (A) KH‐4‐43 treatment yields significantly reduced expression of 20 ovarian cancer hub genes known to be upregulated in ovarian tumours across three ovarian cancer cell lines.^72^ (B) The Hallmark KRAS Signaling Up gene set was significantly enriched in the three KH‐4‐43‐treated ovarian cancer cell lines (A1336, normalized enrichment score [NES] = 1.49, *p*_adjusted_ = .011; A2780, NES = 1.96, *p*_adjusted_ < .0001; SKOV3, NES = 1.60, *p*_adjusted_ = .002). (C) Gene set enrichment analysis reveals consistent patterns of significant pathway enrichment in these cell lines. Color fill indicates *p*_adjusted_ < .05, triangle direction indicates positive (up) or negative (down) enrichment, and the size of the triangles corresponds to the absolute magnitude of the NES. *P*‐values in (B) and (C) are Benjamini–Hochberg adjusted.

To identify transcriptional changes induced by KH‐4‐43 treatment in ovarian cancer cells, we conducted differential expression analysis in three ovarian cancer cell lines, A1336, A2780, and SKOV3. We compared vehicle‐treated cells to cells treated with their respective EC30 dose (the concentration yielding 30% growth inhibition) of KH‐4‐43 (EC30_A2780_ = 3.80 µM, EC30_A1336_ = 3.96 µM, EC30_SKOV3_ = 2.68 µM) (Figure S2A–C). A recent bioinformatic study of normal and cancerous ovarian cells from the Gene Expression Omnibus (GEO) identified 22 hub genes whose simultaneous high expression was associated with poor survival in ovarian cancer, many of which are related to oncogenic pathways.^72^ Our transcriptomic datasets profiled 20 of these hub genes, and we observed that all these genes (except for *CKS1B*, *MCM2*, *RAD51AP1*, and *RCF4* in SKOV3) were significantly downregulated in KH‐4‐43‐treated cells (Figure 4A). We observed a similar gene expression signature reversal of another ovarian cancer hub gene set whose elevated expression was correlated with worse overall survival in patients^73^ (Figure S3). Gene signature reversals such as these have been a promising strategy for drug repurposing in precision medicine.^74^, ^75^ These results suggest that CRL4 inhibition reverses the hub gene signature in ovarian cancer and thereby dampens the proliferative signalling in these cells that likely contributes to their malignancy. One of the hallmarks of cancer is that tumour cells reprogram their signalling and metabolic pathways to allow for aberrant growth,^76^ and the presiding logos of cancer therapy development is that we can more effectively treat cancer by perturbing these evolved, tumour‐specific dependencies. Our observed hub gene signature reversal in ovarian cancer cells, in tandem with the antitumor effects of KH‐4‐43, suggests that disruption of CRL4 activity is sufficient to impede some of the selective advantages employed by these cancerous cells. We also observed that upon KH‐4‐43 treatment, changes occur in the expression of genes related to the mitogen‐activated protein kinase (MAPK) pathway, resulting in the consistent enrichment of the Hallmark “KRAS Signaling Up” gene set (A1336, normalized enrichment score [NES] = 1.49, *p*_adjusted_ < .05; A2780, NES = 1.96, *p*_adjusted_ < .0005; SKOV3, NES = 1.60, *p*_adjusted_ < .005) (Figure 4B, Figure S2D), among other key pathways (Figure 4C, Data S1). The dysregulation of these gene sets serves to further confirm that KH‐4‐43 treatment induces large‐scale disruption of proliferative programs in these cells, thereby limiting their continued malignant growth.

### 2.4 Sixty‐five proteins involved in survival signalling were stabilized in KH‐4‐43‐treated cells

**FIGURE 5:**
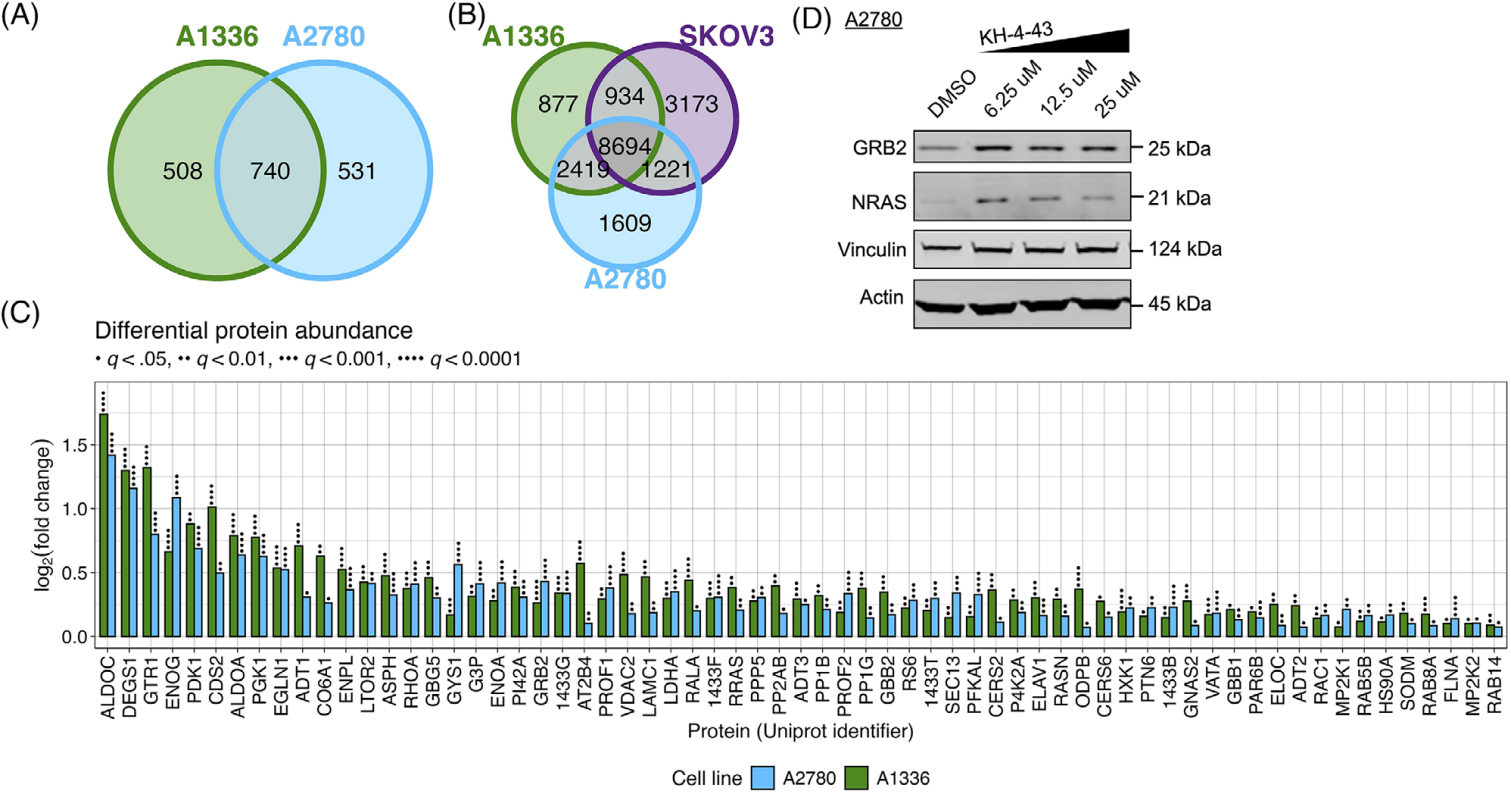
Sixty‐five MAPK‐related proteins were stabilized in KH‐4‐43‐treated cells. Unbiased proteomic profiling of KH‐4‐43‐ and vehicle‐treated A1336 and A2780 cells was conducted, and differential abundances were quantified. Peptides were filtered for those whose log_2_(fold change [FC]) (KH‐4‐43 vs. control) was significantly greater than zero in both A1336 and A2780 (false discovery rate‐adjusted *p*‐value [*q*] < .05). Differential abundance was determined from quadruplicate samples per group per cell line. (A) Venn diagram of proteins that were significantly abundant in KH‐4‐43‐treated A2780 and A1336 cells (log_2_[FC] > 0, *q* < .05). (B) Venn diagram of genes whose expression was not significantly upregulated in KH‐4‐43‐treated A2780, A1336, or SKOV3 cells (log_2_[FC] < 0.5). (C) Bar plot of the log_2_(FC) of the abundance of the 65 proteins that passed the significance filters and met the MAPK inclusion criteria in KH‐4‐43‐treated A2780 and A1336 cells. (D) Western blot of NRAS and GRB2 in A2780 cells treated with KH‐4‐43 at the indicated concentrations for 24 h. Vinculin and actin were used as loading controls.

To nominate proteins that may mediate these signaling responses, we conducted unbiased proteomic profiling of A2780 and A1336 cells treated with vehicle or the cell line‐specific EC30 dose of KH‐4‐43 (EC30_A2780_ = 3.80 µM, EC30_A1336_ = 3.96 µM). We classified proteins that were significantly more abundant in both KH‐4‐43‐treated cell lines (A2780 and A1336) as potential CRL4 targets or proteins that are stabilized by CRL4 substrates. To identify proteins that were more likely to be differentially abundant resulting from KH‐4‐43 treatment rather than inter‐cell line differences or spurious changes, we filtered all detected proteins for those whose log_2_(fold change [FC]) was significantly greater than zero in both A1336 and A2780 (false discovery rate [FDR]‐adjusted *p* < .05), resulting in 740 putative protein targets of CRL4 in these cell lines (Figure 5A). Since increases in protein abundance may also be due to increases in gene expression, we hypothesized that proteins that were most likely to be directly impacted by CRL4 inhibition would be stabilized at the protein level but would not be overexpressed. Subsequently, we classified the genes whose expression in A2780, A1336, and SKOV3 cells were not upregulated by KH‐4‐43 treatment as those with log_2_(FC) < 0.5, resulting in a list of 8,694 genes (Figure 5B). Intersecting the list of 740 proteins with the list of 8,694 genes resulted in a final list of 563 proteins that had the potential to be targets of CRL4. Given the observed upregulation of KRAS signalling‐related genes in KH‐4‐43‐treated cells, we determined how many of these 563 proteins were related to this signalling cascade by filtering for any proteins included in the 25 human Signal Transduction pathways from the Kyoto Encyclopedia of Genes and Genomes^78^, ^79^, ^80^ (Table S3). Of the 1,871 unique genes across these pathway gene lists, 65 of them were included in our list of 563 proteins of interest (Figure 5C).

To confirm the accumulation of these proteins in response to KH‐4‐43 treatment, we selected two proteins that are specifically implicated in activating or propagating MAPK signalling and that carry out unique cellular functions: growth factor receptor bound protein 2 (GRB2) and the GTPase NRAS (whose Uniprot identifier is RASN). GRB2 is an adaptor protein that directly interacts with the cytosolic domain of receptor tyrosine kinases (RTKs) and signals to NRAS through the son of sevenless (SOS) to propagate RTK activation signals through the MAPK cascade.^81^ NRAS is of the Ras gene family and directly facilitates MAPK signalling. Via immunoblotting, we validated that GRB2 and NRAS protein levels increase upon KH‐4‐43 treatment in a dose‐dependent manner (Figure 5D), which may result from enhanced stabilization in the absence of CRL4 activity (i.e., they are direct substrates or are induced by direct substrates). Stabilization of signalling cascade members such as GRB2 and NRAS could be the driving force behind the increased MAPK signalling we observe in KH‐4‐43‐treated cells.

### 2.5 Twenty kinases were predicted to be activated by KH‐4‐43 treatment and may underly the cellular responses to CRL4 inhibition

Due to the limited nature of immunoblotting, our choice of proteins to visualize was partial to specific phosphorylation marks that are indicative of MAPK signalling. To get a more comprehensive and unbiased profile of signaling changes in cells treated with KH‐4‐43, we conducted phosphoproteomic profiling of KH‐4‐43‐ and vehicle‐treated A2780 and A1336 cells (KH‐4‐43 EC30_A2780_ = 3.80 µM, KH‐4‐43 EC30_A1336_ = 3.96 µM). 9,026 phosphopeptides were profiled in A1336 cells (Figure S4A) and 8,671 were profiled in A2780 cells (Figure S4B). To better understand the signalling dysregulation occurring in response to KH‐4‐43 treatment, we utilized the PhosphoSitePlus Kinase Library Enrichment Analysis web tool to infer active kinases from this phosphoproteomic profiling data.^82^, ^83^ This “Kinome Atlas” is the first publicly available computational tool that allows for unbiased prediction of active kinases from pre‐existing phosphoproteomics data by comparing the amino acid context of up‐ and downregulated phosphosites to a library of profiled substrate motifs preferentially modified by 303 human serine/threonine kinases^82^ and 93 human tyrosine kinases (78 canonical and 15 non‐canonical).^83^ Based on the overall up‐ or downregulation of these phosphosites, the Enrichment Analysis tool predicts the magnitude and significance of kinase activity that underlies the observed phosphoproteome changes. We submitted all phosphorylation site flanking regions, fold changes in treated compared with untreated cells, and *p*‐values of all detected phosphopeptides in each cell line to the tool, and we did not filter the phosphorylation sites by significance to maximize input information for the algorithm, which accounts for significance in its predictions. The categorization of these phosphosites is summarized in Table S4.

**FIGURE 6:**
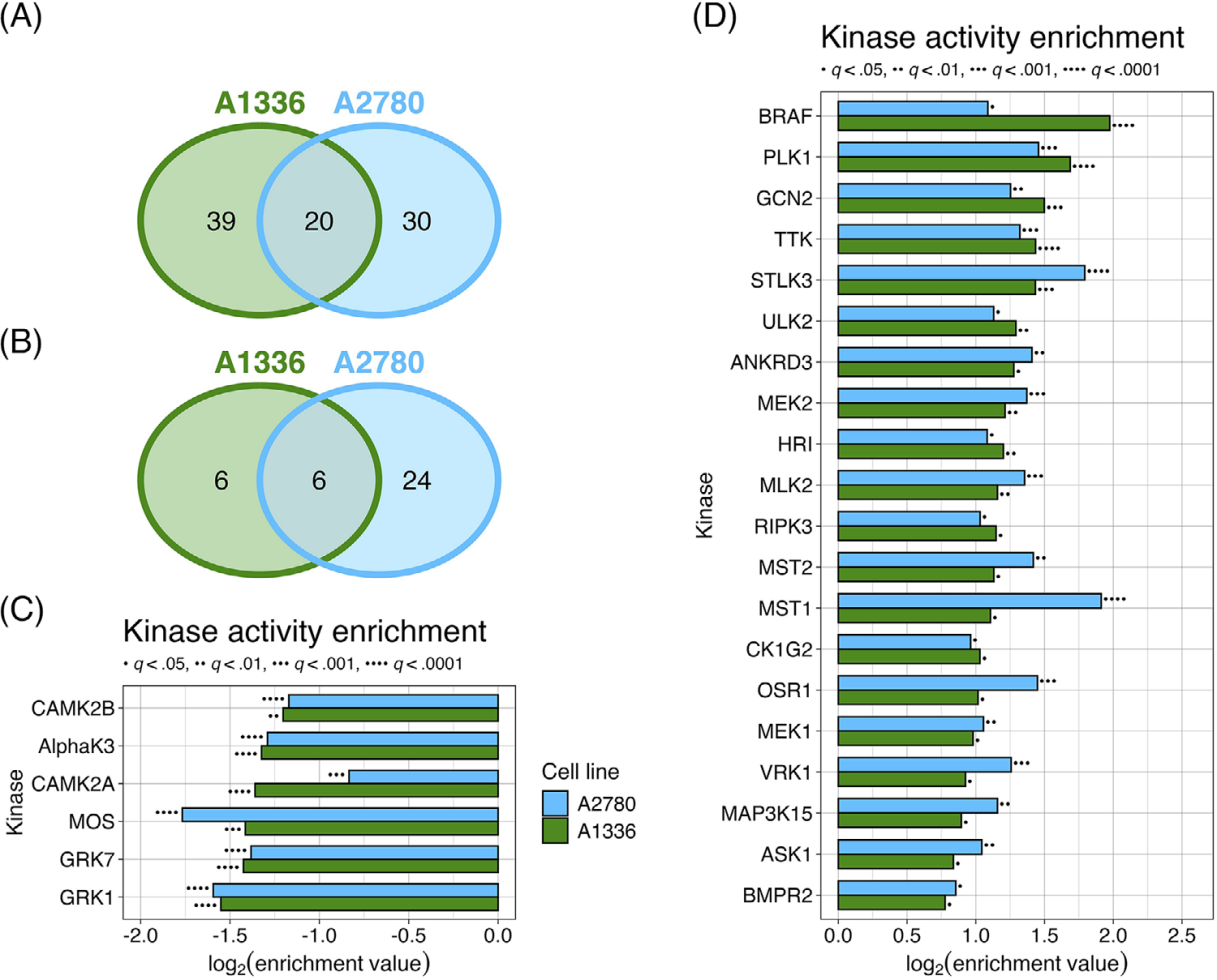
Comparison of dysregulated kinases shared between KH‐4‐43‐treated A1336 and A2780. Differential phosphosite abundances were determined from quadruplicate samples of A1336 and A2780 cells treated with their respective EC30 doses of KH‐4‐43 or vehicle control for 40 h (EC30_A2780_ = 3.80 µM, EC30_A1336_ = 3.96 µM) and were subsequently used to predict dysregulated kinase activity between the treatment and vehicle conditions. The number of unique and shared kinases predicted to be (A) more active or (B) less active in response to KH‐4‐43 treatment are shown. (C) Enrichment values, ordered by decreasing A1336 enrichment values from top to bottom, indicate the extent to which the six shared kinases in (B) were predicted to be less active in KH‐4‐43‐treated cells. Similarly, the data in (D) indicate the extent to which the 20 shared kinases in (A) were predicted to be activated in treated cells. The enrichment factor is a metric derived from relative abundances of phosphorylation motifs in treated and untreated cells, and *q*‐values are Benjamini–Hochberg‐adjusted *p*‐values derived from one‐sided Fisher's exact tests, as described by Johnson et al.^82^ and Yaron‐Barir et al.^83^ MAP3K15 is also called ASK3.

Using the Kinome Atlas, we predicted there to be 59 kinases in A1336 cells and 50 kinases in A2780 cells with significantly upregulated activity (Figure 6A, Figure S4C,D). We also predicted the activity of 12 and 30 kinases to be downregulated in A1336 and A2780 cells, respectively (Figure 6B, Supplementary Figure S4E,F). Of the kinases predicted to be dysregulated, six downregulated and 20 upregulated kinases were shared between A1336 and A2780 cells (Figure 6C,D). BRAF, MEK1, and MEK2 were predicted to be active, which is consistent with the observed upregulation of MAPK signalling in response to KH‐4‐43 treatment. In addition to BRAF, three more of the 20 shared activated kinases were MAPKKKs, which also supports the upregulation of MAPK signalling: apoptosis signal‐regulating kinase 1 (ASK1, encoded by *MAP3K5*), MAP3K15 (also known as ASK3), and mixed lineage kinase 2 (MLK2, encoded by *MAP3K10*). Also predicted to be active were bone morphogenetic protein receptor type 2 (BMPR2), STE20‐like kinase 3 (STLK3), and polo‐like kinase1 (PLK1), all of which are known upstream activators of MAPK signalling. Unc‐51‐like autophagy activating kinase 2 (ULK2) was also predicted to be active, a kinase that is both upstream and downstream of MAPK signalling. Together, this unbiased computational approach supports our observation that MAPK signalling is activated upon CRL4 inhibition.

### 2.6 The combination of CRL4 and MEK inhibition reduces tumor growth in vitro

**FIGURE 7:**
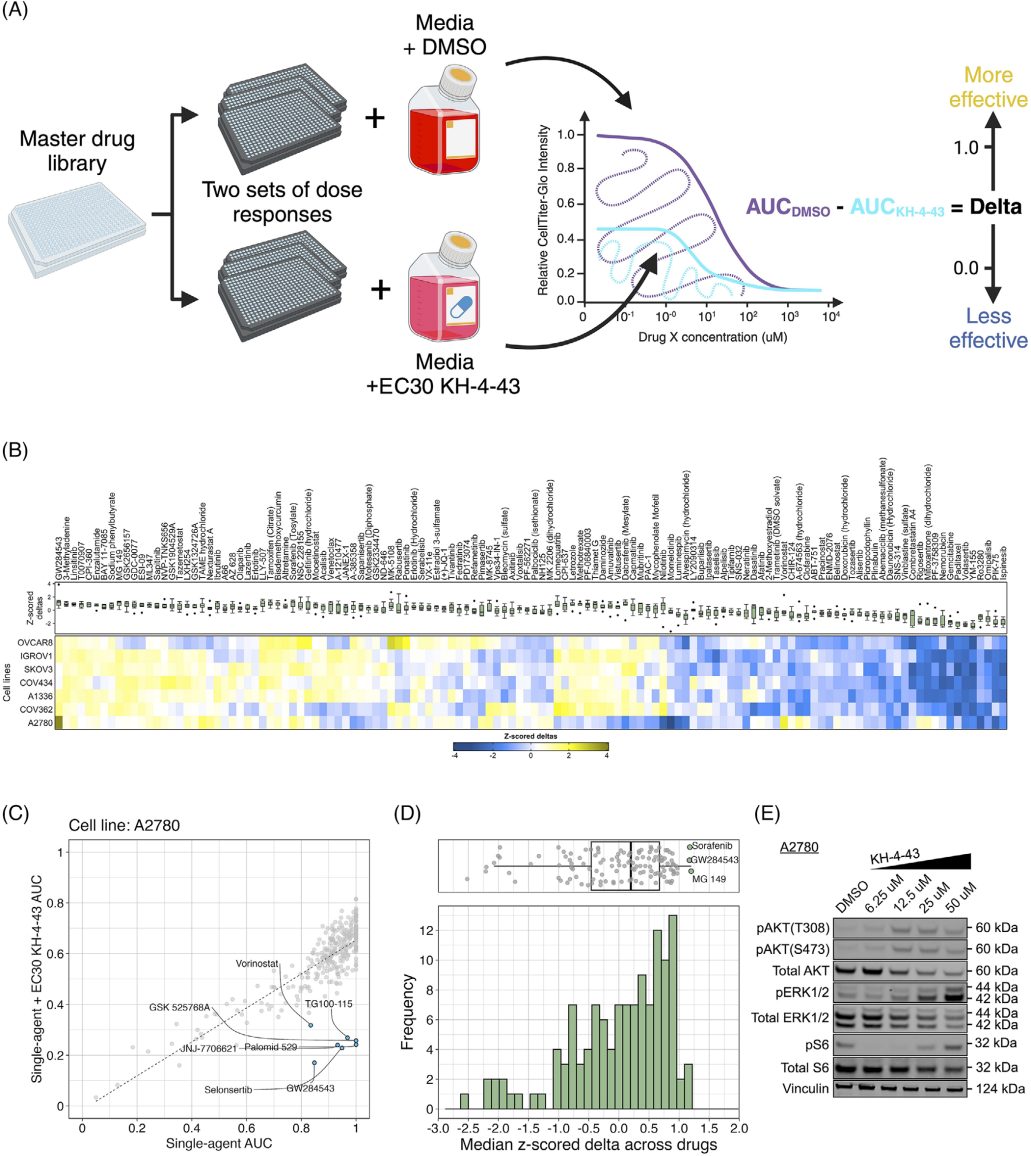
Combination screening reveals potential cooperation between KH‐4‐43 and a MEK inhibitor. (A) Diagram of the high‐throughput combination screening experimental design. AUC values are calculated from dose–response curves fit to triplicate data (created with BioRender.com). (B) Relative effects of KH‐4‐43 drug combination screens across our panel of ovarian cancer cell lines. Scale is mean‐centred z‐scored delta values, computed by normalizing the difference in AUC of our drug library with and without cell line‐specific EC30 dose of KH‐4‐43 across all drugs screened in each cell line (EC30_COV362_ = 1.17 µM, EC30_COV434_ = 4.05 µM, EC30_A1336_ = 4.00 µM, EC30_IGROV1_ = 2.54 µM, EC30_SKOV3_ = 2.60 µM, and EC30_OVCAR8_ = 2.20 µM). Boxplot annotations summarize the per‐drug distribution of z‐scored deltas. Boxes indicated the 25th to 75th percentile (i.e., the inter‐quartile range), thicker midline represented the 50th percentile (i.e., the median), and whiskers indicated the most extreme values within 1.5 times the inter‐quartile range. (C) Exemplary combination screens in A2780 cells revealed cooperativity between KH‐4‐43 and multiple targeted agents. Axes: AUC of our drug library with (*y*) and without (*x*) EC30_A2780_ dose of KH‐4‐43. (D) Distribution of median z‐scored delta values across all drugs screened. For the combination screens, all cell lines were treated with their respective EC30 dose of KH‐4‐43. (E) Western blot in A2780 cells treated with KH‐4‐43 at the indicated concentrations for 24 h. Blot was probed for p‐ERK1/2, p‐AKT at T308 and S473, p‐S6, and totals to examine changes in MAPK signalling over varying doses of KH‐4‐43. Vinculin was used as the loading control. Blots are representative of signalling changes observed in at least two independent experiments.

These data prompted us to investigate potential combination candidate drugs that could enhance the therapeutic efficacy of KH‐4‐43 and help us focus on the impacts of CRL4 inhibition that may regulate its anti‐tumour effects in ovarian cancer. We conducted combination drug screens across a panel of seven ovarian cancer cell lines treated with the cell line‐specific EC30 sensitizing dose of KH‐4‐43 or vehicle control and six‐point dose responses of our full drug panel (Data S2) to identify targetable chemosensitivities induced by KH‐4‐43 treatment and effective therapeutic combinations (EC30_COV362_ = 1.17 µM, EC30_COV434_ = 4.05 µM, EC30_A1336_ = 4.00 µM, EC30_IGROV1_ = 2.54 µM, EC30_SKOV3_ = 2.60 µM, and EC30_OVCAR8_ = 2.20 µM) (Figure 7A–C, Figure S5). We measured the magnitude of the combination effect as the difference in areas under the dose‐response curves (AUCs) between vehicle‐ and KH‐4‐43‐treated cells, which we refer to as “deltas” (Figure 7A,B). A panel of 126 compounds was screened across these seven cell lines (Data S2). Normalization (mean‐centred z‐scoring) of these delta values across all drugs for each cell line allowed for relative comparison of the most effective combinations across the models, with larger z‐scores corresponding to a greater difference in single‐agent and combination AUCs. Examining the distribution of median z‐scores across all compounds, we identified three compounds that seemed to have greater median z‐scores than the other drugs, suggesting they may be the most effective combination partners. One of these agents was a MEK5 inhibitor, GW284543, which had a median z‐scored delta of 1.172 across all cell lines (Figure 7D). With comparable median z‐scored deltas, sorafenib (median = 1.176) and MG 149 (median = 1.201) also show cooperativity with KH‐4‐43. Sorafenib is a multi‐kinase inhibitor with selectivity for c‐RAF and BRAF; MG 149 inhibits the Tip60 histone acetyltranferase, which has been shown to be activated downstream of the MAPK p38^84^ and is an activator of the tumour suppressor p53.^85^, ^86^ Of note, despite previous reports,^87^, ^88^ we did not observe synergy between KH‐4‐43 and the standard of care cisplatin (Figure S6). It is well established that negative feedback mechanisms regulate MAPK activation in healthy cells and that in many cancerous contexts, cells have evolved ways to ignore or bypass these feedback loops, which become potential therapeutic targets.^89^, ^90^, ^91^ Our data suggest that secondary perturbation of growth‐ and survival‐related activation of the MAPK cascade induced by KH‐4‐43 treatment potentiates its antitumor activity, resulting in improved therapeutic efficacy of the drug combinations relative to KH‐4‐43 as a single agent.

**FIGURE 8:**
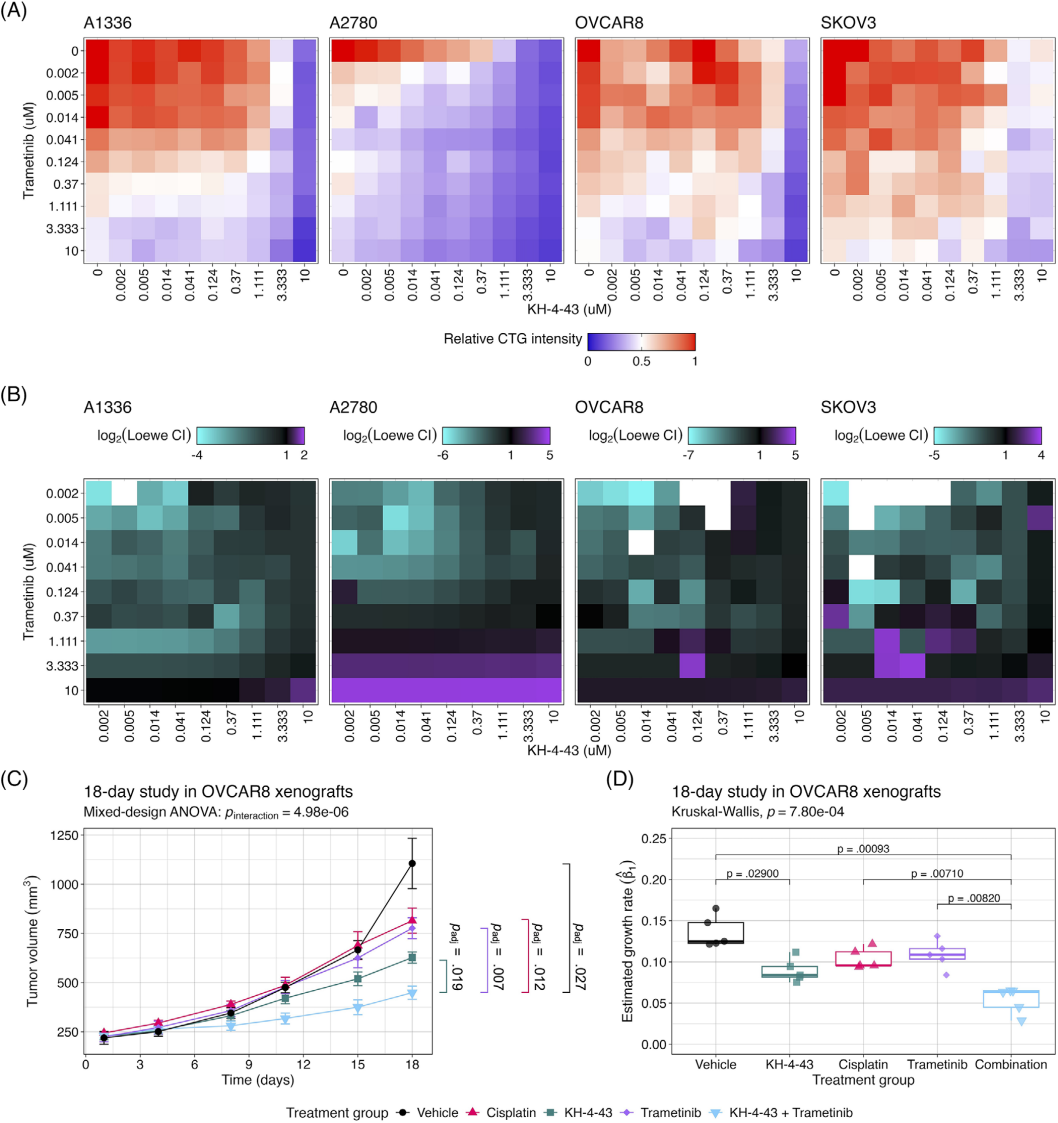
In vitro and in vivo modelling demonstrate the therapeutic potential of combined MEK1/2 and CRL4 inhibition. (A) Using a CellTiter‐Glo (CTG) cell viability assay, variable dosing combination of KH‐4‐43 and trametinib in A1336, A2780, OVCAR8, and SKOV3 cells demonstrates cooperativity between the compounds. The scale is relative to CTG intensity; red = 1 (no death relative to control) and blue = 0 (no viability relative to control). (B) Loewe Additivity combination indices (CIs) were computed across each variable dosing grid screen in (A) and log_2_‐transformed. CI < 1.0 indicates synergy, CI > 1.0 indicates antagonism and CI = 1.0 indicates additivity. Gradient extremes are rescaled on a per‐cell line basis. White tiles indicate that the predicted concentrations of either or both drugs that exhibit the same response, i.e., *A* and/or *B* in Equation (1), were 0.0, resulting in an undefined value indicating there was no response to treatment to compare to *A* and/or *B*. (C, D) OVCAR8 xenografts were generated in female FOXN1^nu^ mice (*n*_group_ = 5). Animals were treated with 50 mg kg^−1^ KH‐4‐43, 1 mg kg^−1^ trametinib, or a combination of KH‐4‐43 and trametinib (daily Monday through Friday) or 3 mg kg^−1^ cisplatin (twice weekly) for 18 days. (C) There was a significant interaction effect of the treatment group and time on tumour volume over the 18‐day treatment period (*p*_interaction_ < .0001, *F* [4, 20] = 16.03, mixed‐design ANOVA of time and treatment main effects). Data represent the mean and standard error of the mean of the number of animals remaining per treatment arm at each timepoint, which was *n*_group_ = 5 for all groups. One‐sided Welch's two‐sample *t*‐tests were used to conduct pairwise comparisons of tumour volumes across treatment arms, *p*‐values were Bonferroni‐corrected for ten total pairwise tests, and only significant comparisons were reported. Day‐18 tumour volumes of the combination‐treated animals were significantly smaller than the vehicle‐treated group (*p*_adjusted_ = .027, *t* [4.55] = 4.98), the cisplatin‐treated group (*p*_adjusted_ = .012, *t* [6.02] = 5.05), the trametinib‐treated group (*p*_adjusted_ = .007, *t* [6.76] = 5.23), and the KH‐4‐43‐treated group (*p*_adjusted_ = .019, *t* [7.79] = 4.07). (D) Linear models were fit to log_2_‐transformed tumour volumes over the 18‐day treatment period. We estimated tumour growth rates as the slope of the best‐fit line, and we see that median growth rates were significantly different across treatment groups (*p* = .001, χ^2^ = 19.02, df = 4, Kruskal–Wallis test), with KH‐4‐43 eliciting a significantly smaller estimated growth rate as compared with vehicle (*p*_adjusted_ = .0292, *t* [7.37] = −4.37, two‐sided Welch's two‐sample *t*‐test). The combination treatment group also had a significantly slower growth rate compared with the vehicle (*p*_adjusted_ < .0001, *t* [7.77] = −7.35, two‐sided Welch's two‐sample *t*‐test). The mean growth rate in the combination treatment was also lower than both the trametinib (*p*_adjusted_ = .008, *t* [7.97] = −5.21, two‐sided Welch's two‐sample *t*‐test) and cisplatin groups (*p*_adjusted_ = .007, *t* [7.49] = −5.51, two‐sided Welch's two‐sample *t*‐test). Pairwise *p*‐values are Bonferroni corrected for the 10 unique pairwise tests conducted in (D). Days is indicative of days post‐treatment initiation. Boxes indicate the 25th to 75th percentile (i.e., the inter‐quartile range), the midlines represent the 50th percentile (i.e., the median), and whiskers indicate the most extreme values within 1.5 times the inter‐quartile range.

We further evaluated the mechanism underlying the observed cooperation between GW284543 and KH‐4‐43 at the signalling level. Considering the effectiveness of the combination across our cell line panel (Figure 7B), the upregulation of the “KRAS Signaling Up” signature in the RNA‐sequencing data (Figure 4B), and the activation of BRAF, MEK1, and MEK2 observed in the phosphoproteomics (Figure 6D), we confirmed dysregulation of MAPK and PI3K signalling at the protein level. Immunoblotting of KH‐4‐43‐teated A2780 cells validated that CRL4 inhibition yields a KH‐4‐43 dose‐dependent increase in flux through the MAPK cascade, as indicated by increased levels of p‐ERK1/2, and revealed increased PI3K signal transduction, as evidenced by concomitant increases in AKT phosphorylation. Levels of p‐S6, a surrogate for mTOR activity and an indicator of both MAPK and PI3K pathway activation, were also shown to increase in response to KH‐4‐43 treatment (Figure 7E). Of note, signalling changes were not uniform across the cell line panel; however, a cell‐specific, dose‐dependent induction of PI3K and/or MAPK signalling was observed in each of the ovarian cancer models tested. In each case, we observed increases in survival signalling that are concordant with the known feedbacks and interplay between the PI3K and MAPK cascades^92^ (Figure S7). This suggests that across the different ovarian models, there is a conserved response to CRL4 inhibition that increases survival signalling through mTOR but that may be specific to the distinct mutational contexts. We further validated the cooperative effects of KH‐4‐43 and GW284543 using our varying dosage grid screening scheme (Figure S8A). These assays indicated consistent synergistic combination indices (CIs) at low doses of both GW284543 and KH‐4‐43, indicating promising interaction between CRL4 and MEK5 inhibition. GW284543 is only used for preclinical research applications, so in order to explore a more translationally relevant combination and leverage our kinase activity predictions (Figure 6D), we subsequently assessed the combination of KH‐4‐43 and trametinib, a widely used, FDA‐approved MEK1/2 inhibitor commonly deployed in combination with other targeted inhibitors^93^ (Figure 8A). Since trametinib targets the same pathway and is already FDA‐approved, we reasoned that this may be a path for faster translation into the clinic. We confirmed cooperativity between KH‐4‐43 and trametinib, with the KH‐4‐43/trametinib combination yielding the greatest overall synergy across all concentrations and cell lines tested (Figure 8B, Figure S8B).

### 2.7 The combination of KH‐4‐43 and trametinib shows therapeutic efficacy in vivo

Given the observed functional cooperativity between KH‐4‐43 and MEK inhibition, we conducted an in vivo study of the combination of KH‐4‐43 and trametinib in cisplatin‐resistant OVCAR8 xenografts in female FOXN1^Nu^ mice (*n*_group_ = 5) over 18 days. Animals were dosed with vehicle control, 3 mg kg^−1^ cisplatin twice weekly or 50 mg kg^−1^ KH‐4‐43 (matching the dose published previously^3^), 1 mg kg^−1^ trametinib,^94^, ^95^, ^96^, ^97^, ^98^ or a combination of KH‐4‐43 and trametinib (daily Monday through Friday) (Figure S9A,B). There was a significant interaction effect of the treatment group and time on tumour volume over the 18‐day treatment period (*p*_interaction_ < .0001, *F* [4, 20] = 16.03, mixed‐design ANOVA of time and treatment main effects) (Figure 8C, Figure S9A). Treatment with the combination also resulted in significantly smaller tumour volumes, highlighting the ability of dual CRL4 and MEK1/2 inhibition to retard tumour growth relative to the standard of care or either agent alone (Figure 8C). Mirroring the approach in our single‐agent trial in OVCAR8 xenografts (Figure 3A,B, Figure S1B), we estimated tumour growth rates in this combination trial between treatment initiation and day 18. We observed that animals treated with the combination of trametinib and KH‐4‐43 exhibited tumour growth rates that were significantly slower than the vehicle (*p*_adjusted_ = .0009, *t* [7.77] = ‐7.25, Welch's two‐sample *t*‐test), cisplatin (*p*_adjusted_ = .0071, *t* [7.49] = −5.51, Welch's two‐sample *t*‐test), and trametinib groups (*p*_adjusted_ = .0082, *t* [7.96] = −5.21, Welch's two‐sample *t*‐test) but not compared with the KH‐4‐43 single agent group (*p*_adjusted_ = .060, *t* [7.87] = ‐3.23, Welch's two‐sample *t*‐test) (Figure 8D, Figure S9C).

We also evaluated the combination of KH‐4‐43 and trametinib in A2780‐derived xenografts (Figure S10). This study corroborated the results from the previous trial with OVCAR8 (*p*_interaction_ < .0001, *F* [4, 20] = 16.03, mixed‐design ANOVA of time and treatment main effects) (Figure S10A) and showed that the combination of KH‐4‐43 and trametinib extended overall survival (*p* = .003, *c* ^2^ = 14.17, df = 3, Mantel‐Cox log‐rank) (Figure S10B) as compared with either drug as a single agent, neither of which showed a significant impact in this more aggressive xenograft model. Immunohistochemical staining of these tumours at the endpoint revealed that combination‐treated tumours had significantly lower levels of p‐S6 staining compared with the vehicle group (*p*_adjusted_ = .046, *t* [6.86] = 3.72, Welch's two‐sample *t*‐test) (Figure S10D,E). There was also a significantly greater number of cleaved caspase‐3 (CC3) foci in trametinib‐ (*p*_adjusted_ = .028, *t* [4.21] = −5.48, Welch's two‐sample *t*‐test), KH‐4‐43‐ (*p*_adjusted_ < .0001, *t* [4] = −49, Welch's two‐sample *t*‐test), and combination‐treated (*p*_adjusted_ = .017, *t* [5.09] = −5.41, Welch's two‐sample *t*‐test) tumors relative to the control group (Figure S10F,G). Ki67 positivity did not differ across treatment groups (Figure S10H). These data from both combination trials indicate that the dual pharmacological inhibition of CRL4 and MEK1/2 outperforms KH‐4‐43 and trametinib as single agents and that these inhibitors cooperate to enhance each other's antitumor activity and may provide benefits in both cisplatin‐sensitive and cisplatin‐resistant ovarian cancer contexts.

## 3 DISCUSSION

Moving forward in the era of precision medicine, it is critical to emphasize the inclusion of emerging therapies in screens of diverse tumour types so that we can identify the optimal patient populations for these agents. By including KH‐4‐43 and 33‐11 in our screening library, we generated functional readouts for these experimental compounds across a panel of two‐dimensional cancer cell lines, diverse in both lineages of origin and genetic background. Through comparison across myriad models, we were able to isolate a statistically significant ovarian cancer sensitivity to CRL4 inhibition. Ovarian cancer is a deadly disease, with the American Cancer Society estimating the 2023 five‐year survival rate to be just 51%.^99^ The most common treatment for ovarian cancers of various subtypes and stages is tumour resection followed by cycles of platinum‐taxane combination therapy, typically carboplatin or cisplatin with paclitaxel or docetaxel,^100^, ^101^ and while some patients remain platinum‐sensitive over many cycles of treatment, the majority become chemoresistant.^102^, ^103^ As a result, many research efforts have pivoted to identifying targeted alternatives or supplements to platinum‐based chemotherapeutics, resulting in the approval of multiple adjuvant therapies for use in relapsed ovarian cancer patients. Specifically, in 2018, the FDA approved bevacizumab, an anti‐VEGF immunotherapy, as a combination therapy with carboplatin and paclitaxel,^104^, ^105^ and in 2020, olaparib, a PARP inhibitor, was approved in combination with bevacizumab for patients with homologous recombination‐deficient cancers.^106^, ^107^ However, even with these recent advances, there have been limited changes to the standard of care for ovarian cancer patients in the last four decades since the FDA first approved cisplatin for the treatment of advanced ovarian cancer in 1978 and paclitaxel in 1992.^108^, ^109^ Many patients with recurrent disease do not have viable therapeutic options, which highlights a need to identify ovarian cancer‐specific dependencies that can be exploited to develop new treatment strategies.

Our findings demonstrate the value of integrating sequencing and functional investigation for identifying potentially effective combination therapy partners and for parsing small‐molecule mechanisms of action. With this approach, we observed a change in flux through the MAPK pathway in response to KH‐4‐43 treatment across in vitro and in vivo ovarian cancer models, which we ascribe to the upregulation of genes within KRAS signalling modules, stabilization of proteins in the MAPK pathway, and increased activity of MAPK‐related kinases like MEK1/2 and BRAF. This increase in signalling likely drives a cellular survival response that is induced by CRL4 inhibition, and this resistance mechanism is mitigated by the inhibition of MAPK signalling via trametinib or other agents targeting the MAPK cascade. By integrating this functional screening with multi‐omic analyses and in vitro and in vivo validation, we were able to show that inhibition of CRL4 (1) elicits a significant antitumor effect in ovarian models, (2) reverses established transcriptomic signatures of ovarian cancer, and (3) induces upregulation of survival signalling mediated by the MAPK cascade, a feedback vulnerability that can be targeted via combination treatment with trametinib. Five ovarian cancer subtypes were represented in our cell line panel (i.e., endometrioid, high‐grade serous, low‐grade serous, serous, and small cell carcinoma) and each of the lines had unique mutational profiles, yet all lines exhibited greater sensitivity to KH‐4‐43 than other tumour types. Our results demonstrate that KH‐4‐43 treatment was effective in ovarian cancer models regardless of mutation status and histological subtype. We also observed sensitivity to KH‐4‐43 in experimentally evolved cisplatin‐resistant cells, thereby suggesting the potential utility of CRL4 inhibition in this key clinical context.

Investigation into more targeted inhibitors of components of the UPS yielded FDA approval of pevonedistat for haematological malignancies. There have been clinical trials assessing pevonedistat in combination with standard‐of‐care therapeutics in advanced solid tumours (NCT00677170,^110^ NCT01862328,^43^ NCT03057366^111^), and these combinations continue to be investigated in non‐haematological contexts (NCT03814005, NCT03323034), though success has been limited. This clinical interest in and preliminary success with pevonedistat supports further investigations into CRL inhibitors as possible therapeutic options for patients with solid tumours, though it has been noted across these trials to cause adverse reactions in some patients. Understanding the differential requirements for E3 ligase activity in various cancers may be an avenue for identifying novel tissue‐specific therapeutic options and complementary agents that target driver mutations. By targeting an individual CRL, we gain specificity and have the potential to see improved efficacy‐to‐toxicity ratios in tumours that are dependent upon a particular E3 without the broad toxicity of agents like proteasome inhibitors or pevonedistat that target broader swaths of the UPS.

Precision oncology workflows like this have the capacity to expand clinical care possibilities for patients with chemoresistant tumours, rare tumour types, and cancers with few therapeutic options. With our growing understanding of the roles of the immune system in tumour management, these workflows could be further augmented to include studies in immunocompetent mouse models or co‐culture models that include immune cells to better understand the complex interplay between emerging therapeutics and anti‐cancer immunity. As new small molecule inhibitors are developed, precision oncology pipelines like ours will be critical for the characterization of their molecular dependencies and effects that will inform their prospects for lead optimization, preclinical testing, and eventual clinical applications. Multi‐omic understanding of the mechanisms by which new drugs exhibit their antitumor effects is critical for innovating not only the treatments that are available but also the way in which patients are profiled to predict their prognoses, responses to therapy, and tumour progression after treatment. With the growing number of targeted therapies available to patients, the unique molecular context of each patient has already become an important component of clinical decision‐making regarding their course of care. The distinct mutations, alterations, and aberrations that each patient has may dictate the extent to which they respond to various therapies, and a thorough grasp of possible drug resistances, interactions, or vulnerabilities will enable more effective prescriptions and improved patient outcomes.

## 4 MATERIALS AND METHODS

### 4.1 In vitro model maintenance

Two‐dimensional cell lines were obtained as indicated in Table S1. All cell lines were maintained in monolayer in the culture media indicated by the suppliers: RPMI 1640 (A2780 and A2780cis cells only; Corning; Roswell Park Memorial Institute Medium; .3 g L^−1^ L‐glutamine, .0053 g L^−1^ Phenol Red•Na) or DMEM (Corning Life Sciences; Dulbecco's Modification of Eagle's Medium; 4.5 g L^−1^ glucose, L‐glutamine, and sodium pyruvate) supplemented with 10% heat‐inactivated fetal bovine serum (Invitrogen) and 1% penicillin‐streptomycin (10,000 Units mL^−1^ and 10,000 µg mL^−1^, respectively) (Invitrogen). As suggested by the distributor, every three passages, the A2780cis growth media was supplemented with 1 µM cisplatin dissolved in phosphate‐buffered saline (PBS). The 541839‐054‐R‐V1 ovarian organoid line was obtained from the National Cancer Institute's Patient‐Derived Models Repository. The base of ovarian organoid culture media is Advanced DMEM/F‐12 (Invitrogen) with 100 ng mL^−1^ noggin (PeproTech), 500 ng mL^−1^ R‐spondin (R&D Systems), 1X B27 supplement (Invitrogen), 10 mM nicotinamide (Sigma‐Aldrich), 1X GlutaMAX‐I (Invitrogen), 1% penicillin‐streptomycin (10,000 Units mL^−1^ and 10,000 µg mL^−1^, respectively), 1% Invitrogen (Invitrogen), 1.25 mM N‐acetylcysteine (Sigma‐Aldrich), 10 µg mL^−1^ primocin (Invitrogen), 1 ng mL^−1^ recombinant human FGF‐basic (PeproTech), 20 ng mL^−1^ recombinant human FGF‐10 (PeproTech), 1 µM PGE2 (R&D Systems), 10 µM SB202190 (Sigma‐Aldrich), 50 ng mL^−1^ recombinant mouse EGF (Invitrogen), 10 µM Y‐27632 ROCK inhibitor (Selleck Chemicals), 500 nM A‐38‐01 (R&D Systems), and 10 ng mL^−1^ NRG (PeproTech). Organoids were maintained as 100‐µL dots of 1:2 ovarian culture media‐to‐Matrigel (Corning Life Sciences), with each dot containing approximately 10^5^ cells upon plating. Four to six dots were plated in each well of a six‐well plate, and after 30 min of setting at 37°C, 2 mL of ovarian organoid media was added to each well. Organoids were checked every three days and expanded as needed. All cells, two‐ and three‐dimensional, were maintained in a 37°C humidified incubator with 5% CO_2_.

### 4.2 Functional genomics pipeline (high‐throughput screening)

Lab automation was used for cell plating, drug dosing, plate imaging, and endpoint readout. The custom drug library for functional screening was purchased from MedChemExpress with all compounds dissolved in dimethyl sulfoxide (DMSO) (Data S2–3). Compounds ordered in larger quantities were made into 10 mM stock solution (in DMSO) and stored at −80°C according to manufacturer instructions. KH‐4‐43 and 33‐11 syntheses were previously reported,^3^ re‐synthesized at BioDuro, and stored in DMSO at −80°C.^3^ 10 mM cisplatin in DMSO (Medical and Biological Laboratories International protocol) was made fresh for all experiments except for the 14‐day OVCAR8 study for which 10 mM cisplatin was made fresh in dimethylformamide (MedCemExpress protocol). The automated platform consists of a Janus Liquid Handling System (Revvity), an Echo 650 Acoustic Sampler (Beckman Coulter), an Automated Incubator with a 500‐plate capacity (Liconic), an Opera Phenix High Content Screening System (Revvity), a CLS High Content Imaging System (Revvity), a Nivo Plate Reader (Revvity), and an Automated Microplate Centrifuge (Agilent). Two‐dimensional cell lines were seeded on 348‐well plates (Revvity) at a density of ∼1,500 cells per well (depending on cell line‐specific doubling time) and grown for 24 h, at which point the media was refreshed and the wells were dosed with all compounds in the drug library in six‐point, 1:3 dilution dose responses (maximal dose of 10 µM and minimal dose of .04 µM with vehicle backfilling to maintain constant vehicle volume in all wells) with 18‐vehicle (DMSO) wells on each 384‐well plate. All compounds in the drug library were dosed in triplicate. The outer two rows and columns of wells contained media but were not seeded to provide a buffer against edge effects of media evaporation. For combination dose response screens, replacement media contained the sensitizing concentration (the concentration yielding 30% maximal response, EC30) of the compound of interest. After 72 h of treatment, cells were treated with CellTiter‐Glo Luminescent Cell Viability reagent (Promega) to assess cell viability at the endpoint according to the standard product protocol. Cell viability for each dosed well was normalized against the per‐plate vehicle well average cell viability, yielding our viability metric of “Relative CTG Intensity”. For all dose–response curves, EC30 values were computed from curves fit using the four‐parameter logistic starter function of the drc R package (v3.0‐1),^112^ and area under the dose–response curve (AUC) values were computed using the PharmacoGx R package (v3.2.0).^113^ For combination grid screens, each of the two drugs was varied in a nine‐point 1:3 dilution dose response (maximal dose of 10 µM and minimal dose of .0015 µM) and a true 0 µM well with vehicle only. Two grids were plated per 384‐well plate, and each combination was run in triplicate. Cell viability was normalized to the vehicle‐only wells, and the triplicates were averaged to produce heatmaps. Loewe Additivity combination indices (CI_Loewe_) were calculated using Equation (1)^114^ and visualized using ggplot2 (v3.4.3).^115^

(1) $$C\;I_{\mathrm{Loewe}}=\frac{a}{A}+\frac{b}{B},$$

where *a* is the concentration of drug A in the combination, *A* is the predicted concentration of drug A required to achieve the same effect as the combination, *b* is the concentration of drug B in the combination, and *B* is the predicted concentration of drug B required to achieve the same effect as the combination.

### 4.3 Dose–response validation

Two‐dimensional cell lines were manually plated in triplicate in black, clear bottom, 96‐well tissue culture plates (Thermo Scientific) at a density of 4,000 cells per well. Cells were manually dosed in the same way as described above. At endpoint, plates were treated as described above and were read on a Tecan Infinite 200 PRO microplate reader at 565 nm. Normalization, curve fitting, and computations were conducted as described above.

### 4.4 Western blotting

Cells were seeded in six‐well plates at a density of approximately 300,000 cells per well or 6‐cm plates at a density of approximately 10^6^ cells per plate. Approximately 24 h after plating, cells were treated with the appropriate dose of the compound of interest or vehicle control with complete culture media replacement. The vehicle concentration was consistent across all conditions tested in each experiment. After a 24 h dosing period, cells were rinsed with cold PBS, and plates were put on ice. Cells were lysed in NP‐40 Cell Lysis Buffer (Invitrogen) (50 mM Tris, pH 7.4, 250 mM NaCl, 5 mM EDTA, 50 mM NaF, 1 mM Na_3_VO_4_, 1% detergent, .02% NaN_3_) containing 1X Halt Protease and Phosphatase Inhibitor cocktail (Thermo Scientific) and mechanically harvested with a cell lifter. Protein was isolated in the supernatant after centrifugation at 10,000 × *g* for 10 min at 4°C. Protein concentration was quantified using the Pierce BCA Protein Assay Kit (Thermo Scientific). Protein was immunodetected after SDS‐polyacrylamide gel electrophoresis on NuPAGE 4–12% gradient, Bis‐Tris, 1.0‐mm pre‐cast gels (Invitrogen). Protein was transferred using the iBlot dry blotting system (Invitrogen) following a three‐step protocol: 1.5 min at 20 V, 4.5 min at 23 V, and 3.0 min at 25 V. Membranes were blocked with 5% bovine serum albumin (BSA) (Thermo Scientific) in tris‐buffered saline with Tween 20 (TBST) for 15 min to 1 h at room temperature. All primary antibodies were diluted in 1% BSA in TBST at a dilution of 1:1,000 unless otherwise stated below. Membranes were incubated with the indicated primary antibodies at 4°C overnight, washed for 10 min three times with 1X TBST, incubated with secondary antibodies (1:10,000) at room temperature for 1 hour, and washed again for 10 min three times with 1X TBST. Membranes were visualized using the Odyssey DLx Infrared Imaging System and processed using ImageStudio (v4.0.21) and EmpiriaStudio (v2.2.0.141) (both from Li‐Cor Biosciences). Dose–response western blot experiments were either conducted in duplicate or were expanded from experiments that demonstrated similar changes with a single dose (i.e., KH‐4‐43 EC30 vs. vehicle).

The following primary antibodies were used: anti‐β actin (Cell Signaling Technology, #3700), anti‐vinculin (Cell Signaling Technology, #13901), anti‐phosphorylated Thr202/Tyr204 ERK1/2 (p‐pERK1/2) (Cell Signaling Technology, #4370), anti‐ERK1/2 (p44/42 MAPK) (Cell Signaling Technology, #9102), anti‐phosphorylated Thr308 AKT (Cell Signaling Technology, #13038), anti‐phosphorylated Ser473 AKT (p‐AKT473) (Cell Signaling Technology, #4060), anti‐AKT (pan) (Cell Signaling Technology, #2920), anti‐phosphorylated S6 ribosomal protein (p‐S6) (Cell Signaling Technology, #5364), anti‐S6 ribosomal protein (Cell Signaling Technology, #2317), anti‐N‐ras (Thermo Scientific, #703435), and anti‐GRB2 (Cell Signaling Technology, #3972). The following secondary antibodies were prepared, aliquoted, and stored in the dark at −20°C following the manufacturer's protocols: IRDye 680RD Goat anti‐Rabbit IgG (H+L) (Li‐Cor, #926‐68071), IRDye 800CW Donkey anti‐Mouse IgG (H + L) (Li‐Cor, #926‐32212), IRDye 800CW Donkey anti‐Rabbit IgG (H + L) (Li‐Cor, #926‐32213), and IRDye 680RD Goat anti‐Mouse IgG (H+L) (Li‐Cor, #926‐68070).

### 4.5 RNA‐sequencing

Cells were seeded in six‐well plates at a density of approximately 300,000 cells per well. After 24 h, cells were treated in triplicate with the EC30 of KH‐4‐43 or DMSO control with complete culture media replacement. After 18 h of dosing, cells were rinsed with cold PBS, harvested via cell lifter, and pelleted by centrifugation at 400 × *g* for 5 min at 4°C. Pellets were resuspended in 50 µL buffer RLT (Qiagen) with 1% β‐mercaptoethanol (Thermo Scientific) and flash‐frozen in liquid nitrogen. Samples were stored at −80°C and submitted to Genewiz from Azenta Life Sciences for standard RNA sequencing (approximately 20–30 million reads per sample and poly‐A selection for rRNA removal) with their standard RNA analysis package that includes trimming, mapping, differential gene expression analysis, and preliminary Gene Ontology^116^, ^117^ enrichment. Differential gene expression analysis was conducted using the DESeq2 R package (v1.20),^77^ and *p*‐values were computed using Wald tests and adjusted using the Benjamini–Hochberg (BH) method to decrease the false discovery rate.^118^ Gene set enrichment analysis (GSEA) was conducted using MSigDB Hallmark gene sets^119^, ^120^, ^121^ with the fgsea R package (v1.24.0).^122^ All adjusted *p*‐values associated with GSEA analyses were corrected using the BH procedure.^118^

### 4.6 Global proteomics and phosphoproteomics

We profiled two of our ovarian cancer cell lines, A2780 and A1336, that had been treated with KH‐4‐43 or vehicle control. A2780 and A1336 cells were treated with the EC30 dose of KH‐4‐43 or DMSO vehicle in quadruplicate for 40 h to maximize the magnitude of proteome/phosphoproteome change, harvested as described previously, and cell pellets were snap‐frozen in liquid nitrogen. Samples were submitted to the New York University Proteomics Laboratory where they were processed and submitted for liquid chromatography‐tandem mass spectrometry (LC‐MS/MS). All samples were processed in parallel in a single batch. Briefly, cells were lysed by sonication in a 5% sodium deoxycholate (SDC) denaturing buffer and incubated at 90°C. Samples were diluted to 1% SDC, and proteins were trypsin‐digested in solution. Peptides were quantitated both separately (i.e., label‐free) and pooled (i.e., labelled). For separate quantitation, samples were run by LC‐MS/MS directly after protein digestion. Data were acquired in data‐independent mode (DIA) for label‐free quantification (LFQ) and subsequently analyzed using Spectronaut. For the pooled processing, samples were tandem mass tagged (TMT) with TMTPro isobaric tags, pooled, fractionated by high‐pH RP C18 offline HPLC chromatography (*n*_fractions_ = 32), and analyzed in data‐dependent mode for MS2‐based TMT quantification with MaxQuant. Of note, the Proteomics Laboratory identified a significant drop in protein intensity for one of the KH‐4‐43‐treated replicates of A2780 cells, which they identified as due to a technical error. Dr. Evgeny Kanshin conducted coefficient of variation analyses with and without the outlier sample, validating and censoring the outlier in downstream analyses.

For phosphoproteomic profiling, peptides phosphorylated at serines, threonines, and tyrosines (pSTY) were enriched via Fe‐IMAC and quantitated via DIA for LFQ using Spectronaut. Since phosphopeptides comprise ∼1% of total peptides in these types of samples, only one enrichment and one analysis were conducted for each sample. Using the Kinase Library Enrichment Analysis (v0.0.10)^82^ tool from PhosphoSitePlus^123^ (www.phosphosite.org/kinaseLibraryAction), differentially active kinases were inferred from the phosphorylated peptide data in the KH‐4‐43‐treated and control‐treated groups. The input sequence data was formatted following the “central position” option where phosphorylation sites (S, T, or Y) were flanked by seven amino acids, yielding a 15‐amino acid sequence. Underscores, “_”, indicated truncations. Statistical evaluation of global and phosphoproteomic data was conducted by Dr. Kanshin of the New York University Langone Proteomics Laboratory. Briefly, the significance of fold changes of protein and phosphopeptide abundances was evaluated using two‐sample *t*‐tests and adjusted using the permutation‐based false discovery rate correction method.

### 4.7 Xenograft studies

Since we are focusing on ovarian cancer, only female mice were used. To develop xenografts, ovarian tumour cells grown in DMEM or RPMI (as described in *In vitro model maintenance* methods) were mixed with Matrigel (Corning Life Sciences) in a 1:1 ratio. 100 µL of the 1:1 homogenate containing approximately 10^6^ cells were subcutaneously injected unilaterally in the right flanks of 8–10 weeks old, female, FOXN1^nu^ mice (Jackson Labs). When the tumour diameter reached approximately 0.65 cm, mice were randomly grouped into each treatment arm and treatment was initiated. Doses were administered via intraperitoneal injection (KH‐4‐43 and cisplatin) or gavage (trametinib). Animals were monitored three times per week for indications of adverse effects of treatment. Tumour volumes were calculated by Equation (2).^124^ Upon endpoint, tumour tissue was excised and fixed in 4% paraformaldehyde in PBS. Primary readouts for the long‐term studies were overall survival and tumour burden. Statistical tests for in vivo experiments were conducted using base R, the ggpubr R package (v0.6.0),^125^ sigminer (v2.2.2),^126^ and GraphPad Prism 8. For the short‐term studies, under the assumption of exponential growth, per‐animal estimated tumour growth rates (${\hat{\beta }}_{1}$) were determined via least‐squares simple linear regression modelling of log_2_‐transformed tumour volume over time (Equation [ 3]).^69^

(2) $$\mathrm{Volume}\;=\frac{4}{3}\;\pi \times \frac{L}{2}\times \frac{W}{2}\times \frac{L+W}{4}\times 10^{3},$$

where *L* and *W* are perpendicular measures of tumour diameter in centimetres and volume in cubic millimetres.

(3) $$\mathrm{lo}g_{2}\;\left(\mathrm{tumor}\;\mathrm{volume}\right)=\beta_{0}+\beta_{1}\times t+\varepsilon ,$$

where coefficients *β_0_* and *β_1_* represent the initial log_2_‐transformed tumour volume and linear growth rate, respectively; *t* is time; and *ε* is the error term.

### 4.8 Immunohistochemistry

Tumour tissue samples were formalin‐fixed and paraffin‐embedded (FFPE), sectioned on Superfrost Plus Microscope Slides, and hemotoxylin and eosin stained in the Histology Shared Resource in the Department of Oncological Sciences at the Icahn School of Medicine at Mount Sinai and the Center for Translational Pathology at Weill Cornell Medicine. Immunohistochemistry was carried out on FFPE tumour tissue sections using an automated LeicaBond RX immunostaining instrument (Leica Biosystems) following validated manufacturer protocols. Slides were deparaffinized and washed using heated Bond Dewax Solution (Leica Biosystems, #AR9222) and 1X Bond Wash Solution (Leica Biosystems, #AR9590), respectively. Citrate‐based Bond Epitope Retrieval 1 Solution (Leica Biosystems, #AR9961) was used for 30 min epitope retrievals. After blocking in 3–4% v/v hydrogen peroxide for 5 min, slides underwent DAB staining with the Bond Polymer Refine Detection Kit (Leica Biosystems, #DS9800). Slides were incubated for 1 h at room temperature with primary antibodies diluted in Bond Primary Antibody Diluent (Leica Biosystems, #AR9352). Secondary antibodies (mouse and rabbit) used were from the Bond Polymer Refine Detection kit (Leica Biosystems, #DS9800) or the Bond Polymer Refine Red Detection kit (Leica Biosystems, #DS9390). The following primary antibodies and dilutions were used: anti‐Ki67 (1:100, Abcam, #ab16667), anti‐p‐S6 (1:1000, Cell Signaling Technology, #5364), and anti‐cleaved caspase 3 (1:200, Cell Signaling Technology, #9664). Slides for the OVCAR8 combination study were scanned using an Aperio digital pathology slide scanner (Leica Biosystems) and images were viewed and analyzed using the Aperio eSlide Manager system (Leica Biosystems). Slides for the A2780 xenograft study were scanned using a Nano Zoomer S60 (Hammamatsu Photonics, #000026) and images were viewed and analyzed using the NDP.view2 software (v2.9.29, Hamamatsu Photonics, #U12388‐01). Immunohistochemically stained slides were evaluated by a study pathologist based on vital tumour cells, excluding tumour‐infiltrating lymphocytes. The staining was scored using a semi‐quantitative technique (H‐score).^127^

### 4.9 Summary of statistical analyses

Dose responses and grid screens were performed in triplicate, RNA‐sequencing samples were submitted in triplicate, and proteomics samples were submitted in quadruplicate. Multiple testing corrections were conducted for datasets for which two or more analyses were conducted, as noted in the respective methods sections and summarized below. *P*‐values and adjusted *p*‐values less than .05 were considered significant. All statistics were conducted using either GraphPad Prism 8 or R statistical language (v4.3.2),^128^, ^129^ whose base components include the stats package (v4.3.2). For the 14‐day OVCAR8 xenograft study, mixed‐design ANOVAs with a between‐subjects factor (also called repeated measures ANOVAs with a between‐subjects factor) were used to evaluate the interaction between time and treatment on tumour volume using the ez R package (v4.4‐0).^130^ For xenograft studies evaluating overall survival where animals had differing lifespans prior to endpoint, effects of the treatment group and time on the dependent variable were modelled using linear mixed‐effect models fit by maximum likelihood with the lmerTest R package (v3.1‐3)^131^ and significance was tested with type III ANOVAs with Satterthwaite's approximation of the degrees of freedom using the stats R package. For the overall survival xenograft studies, a significant difference between the survival curves across treatment groups was assessed with Mantel‐Cox log‐rank tests in GraphPad Prism 8. In cases where there were distributions from more than two groups to compare, nonparametric Kruskal‐Wallis tests (stats R package) were conducted. Pairwise comparisons were evaluated using Welch's two‐sample *t*‐test, and *p*‐values were adjusted using the Bonferroni multiple‐comparison correction method to adjust for familywise error. Significance of differential transcriptomics was conducted using the DESeq2 R package (v1.20) as previously described; briefly, *p*‐values for differential expression of individual genes are computed via Wald tests and corrected using the BH method.^132^ GSEA was conducted with the fgsea R package (v1.24.0), and *p*‐values were BH‐adjusted according to default settings.^122^ Phosphoproteomic motifs were submitted to the Enrichment Analysis tool of The Kinase Library (v0.0.10)^82^, ^83^ available from PhosphoSitePlus^123^ (www.phosphosite.org/kinaseLibraryAction), whose algorithm conducts one‐sided Fisher's exact tests to evaluate the significance of kinase activity prediction and corrects *p*‐values using the BH method.

## AUTHOR CONTRIBUTIONS

Sally E. Claridge, Zhen‐Qiang Pan, and Benjamin D. Hopkins developed the project. Sally E. Claridge, Shalini Nath, Nile M. Rizvi, Lamberto De Boni, Eric Park, Richard Farias, Genesis Lara Granados, Matthew Hauesgen, Julie‐Ann Cavallo, Chantal Pauli, and Benjamin D. Hopkins all contributed meaningfully to the development of the Functional Genomics Pipeline (FGP) including but not limited to workflow conceptualisation, method development, validation, and data processing and management. Kenneth Wu, Khoi Q. Huynh, Peng‐Jen Chen, Fred R. Hirsch, Juan Miguel Mosquera, Olivier Elemento, Robert J. DeVita, Zhen‐Qiang Pan, and Benjamin D. Hopkins provided resources and materials, e.g., cell lines, compounds, and facilities. Sally E. Claridge, Shalini Nath, Richard Farias, Anneliese Baum, and Benjamin D. Hopkins performed experiments outside the main FGP. Shalini Nath and Benjamin D. Hopkins conducted murine trials. Ruben Fernandez‐Rodriguez optimised and conducted immunohistochemical staining of tumour tissues, and Chantal Pauli and Eda Nur Kozan scored the resulting immunohistochemical images. Evgeny Kanshin and Beatrix Ueberheide conducted mass spectrometry and peptide quantification. Sally E. Claridge and Benjamin D. Hopkins analysed the data and wrote the manuscript, and all other authors provided reviews and revisions. All authors have read and agreed to the submitted version of the manuscript.

## CONFLICT OF INTEREST STATEMENT

B.D.H. is a founder and consultant for Faeth Therapeutics. The Hopkins Laboratory has received support from Faeth Therapeutics, Jazz Pharmaceuticals, and Novartis.

## CODE AVAILABILITY

All code written and utilized in the current study is available from the corresponding author upon reasonable request.

## ETHICS STATEMENT

All animal work conducted for this publication is under IACUC‐approved protocols (2019‐0018 at the Icahn School of Medicine at Mount Sinai and 2018‐0038 at Weill Cornell Medicine), which are renewed annually.

## Supporting information



Supporting information

Supporting information

Supporting information

Supporting information

Supporting information

Supporting information

Supporting information

Supporting information

Supporting information

Supporting information

Supporting information

Supporting information

Supporting information

Supporting information

## Data Availability

The datasets generated and/or analyzed during the current study are available from the corresponding author upon reasonable request.
