# Supplementary figures and images for: Functional genomics pipeline identifies CRL4 inhibition for the treatment of ovarian cancer
Source: Clin Transl Med. 2025 Jan 24;15(2):e70078. doi: 10.1002/ctm2.70078 (PMC11761363; doi:10.1002/ctm2.70078)

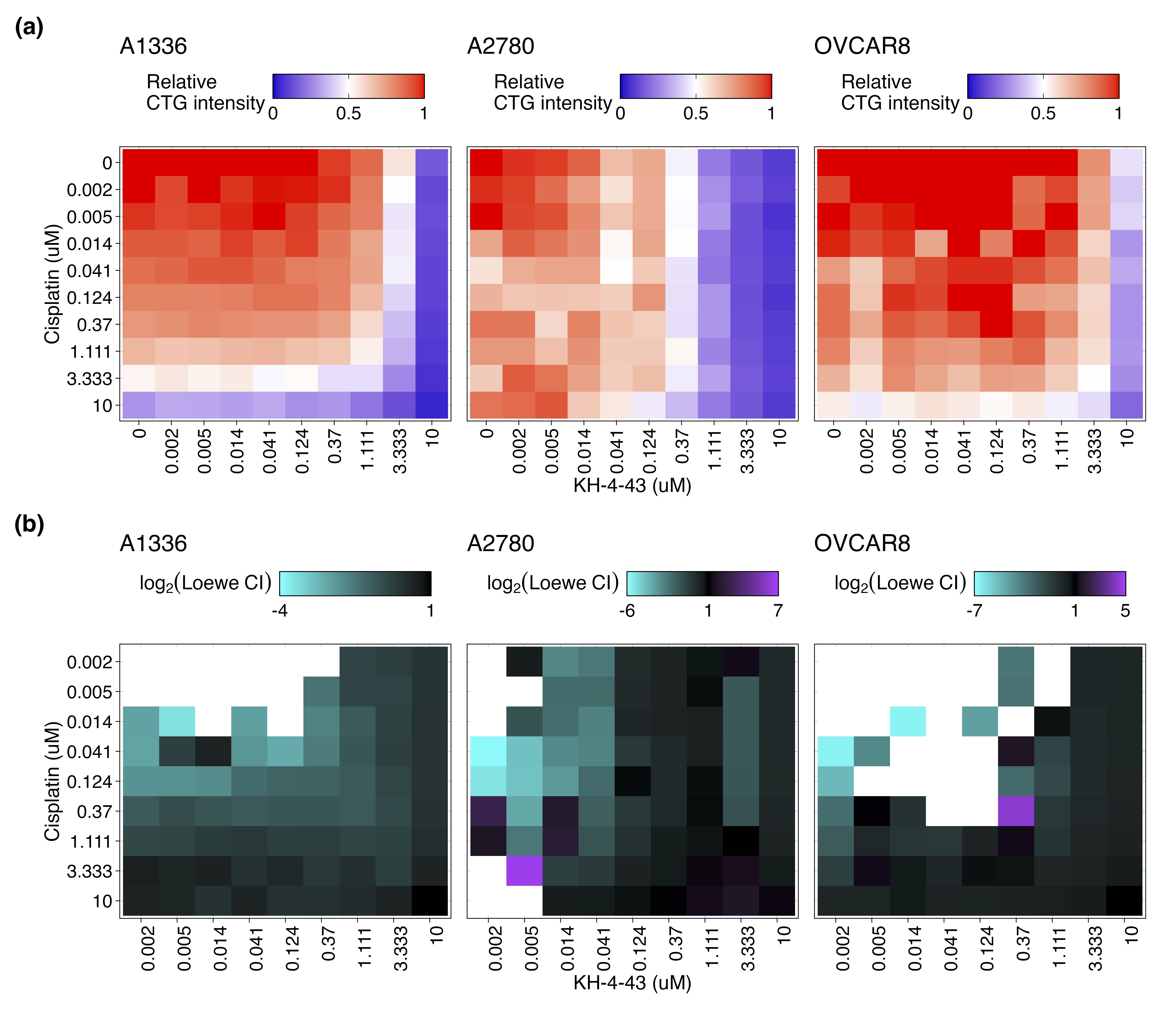

Supplement: Supplementary file 1 — Supporting information [file CTM2-15-e70078-s006.jpg]

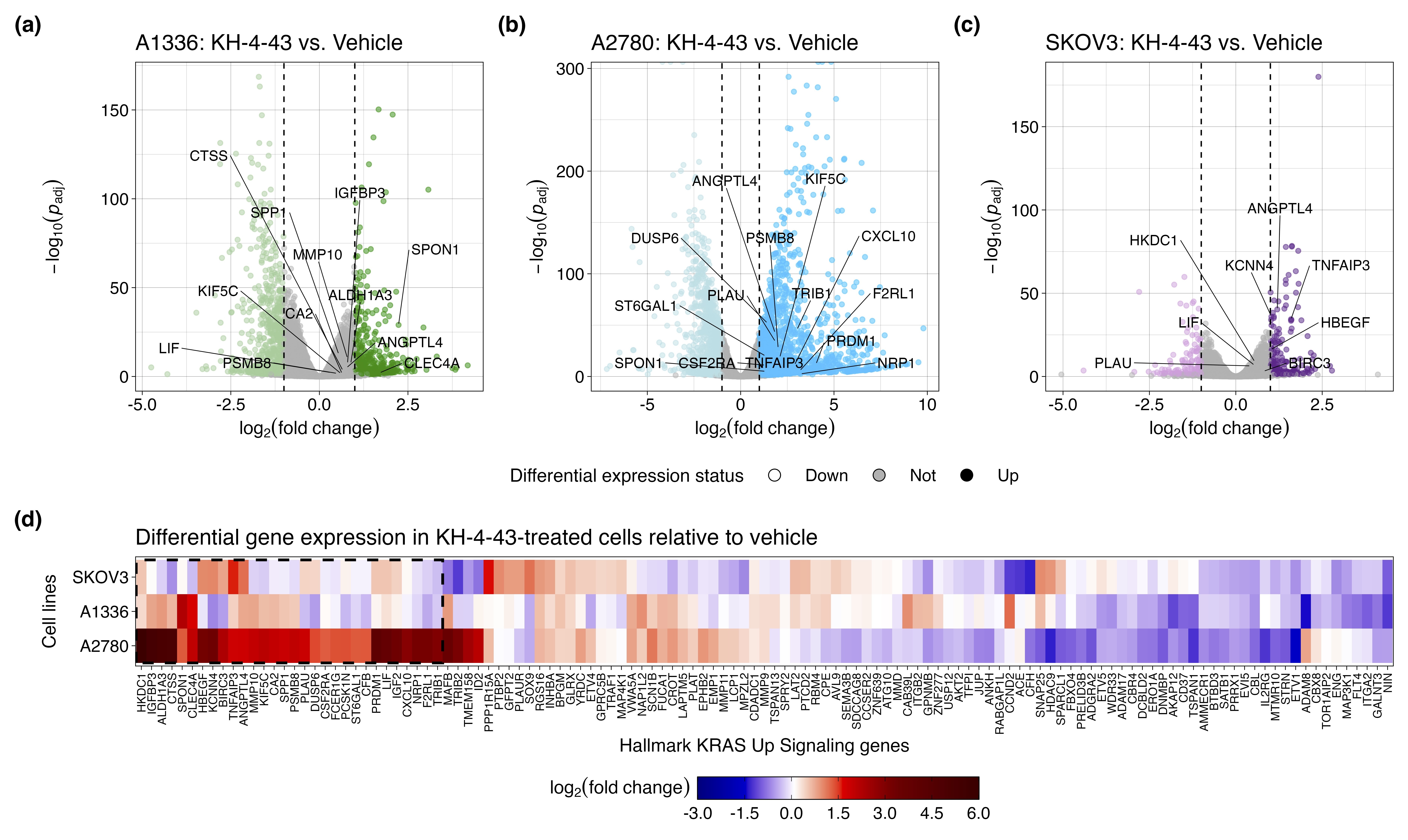

Supplement: Supplementary file 2 — Supporting information [file CTM2-15-e70078-s012.jpg]

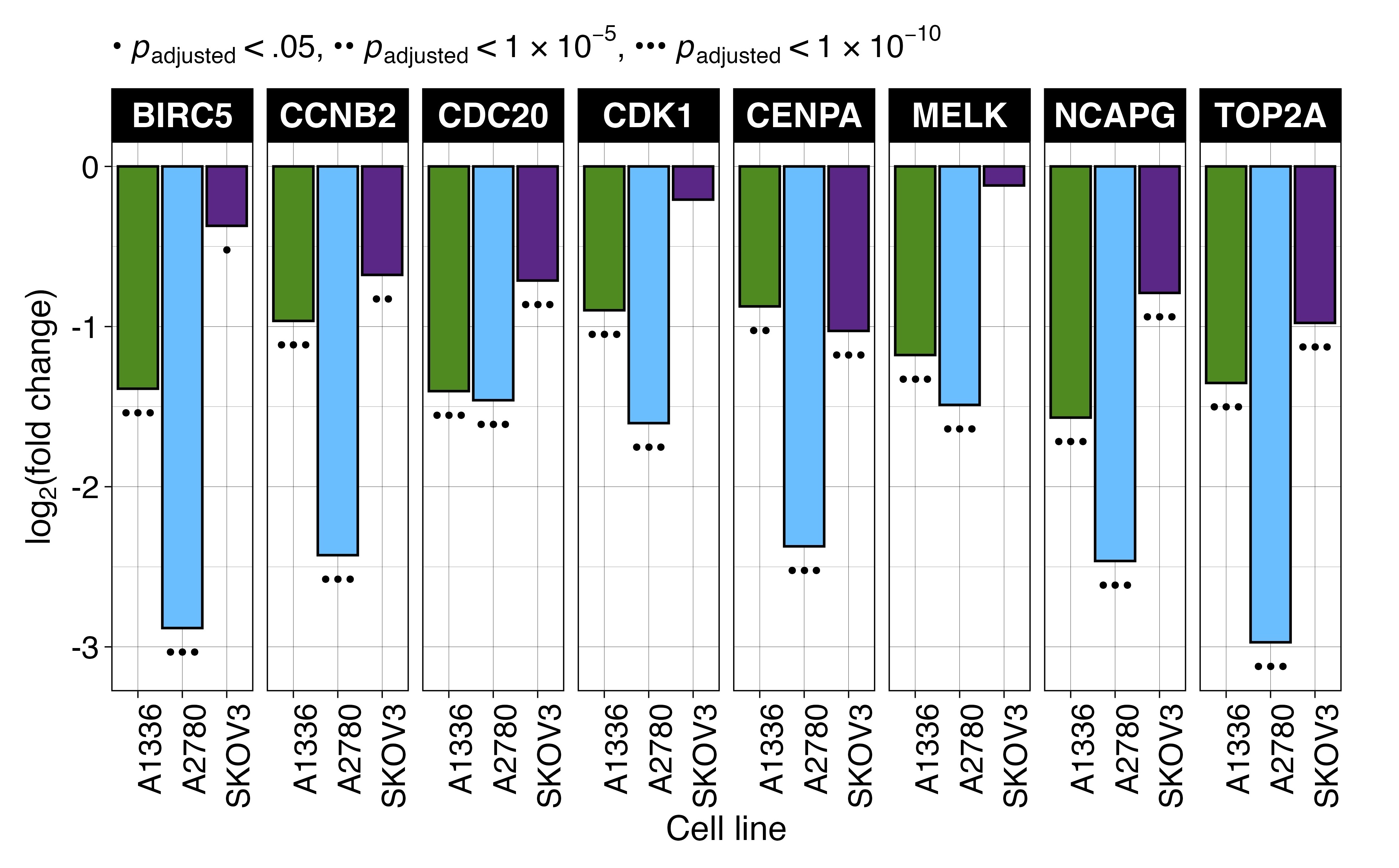

Supplement: Supplementary file 3 — Supporting information [file CTM2-15-e70078-s003.jpg]

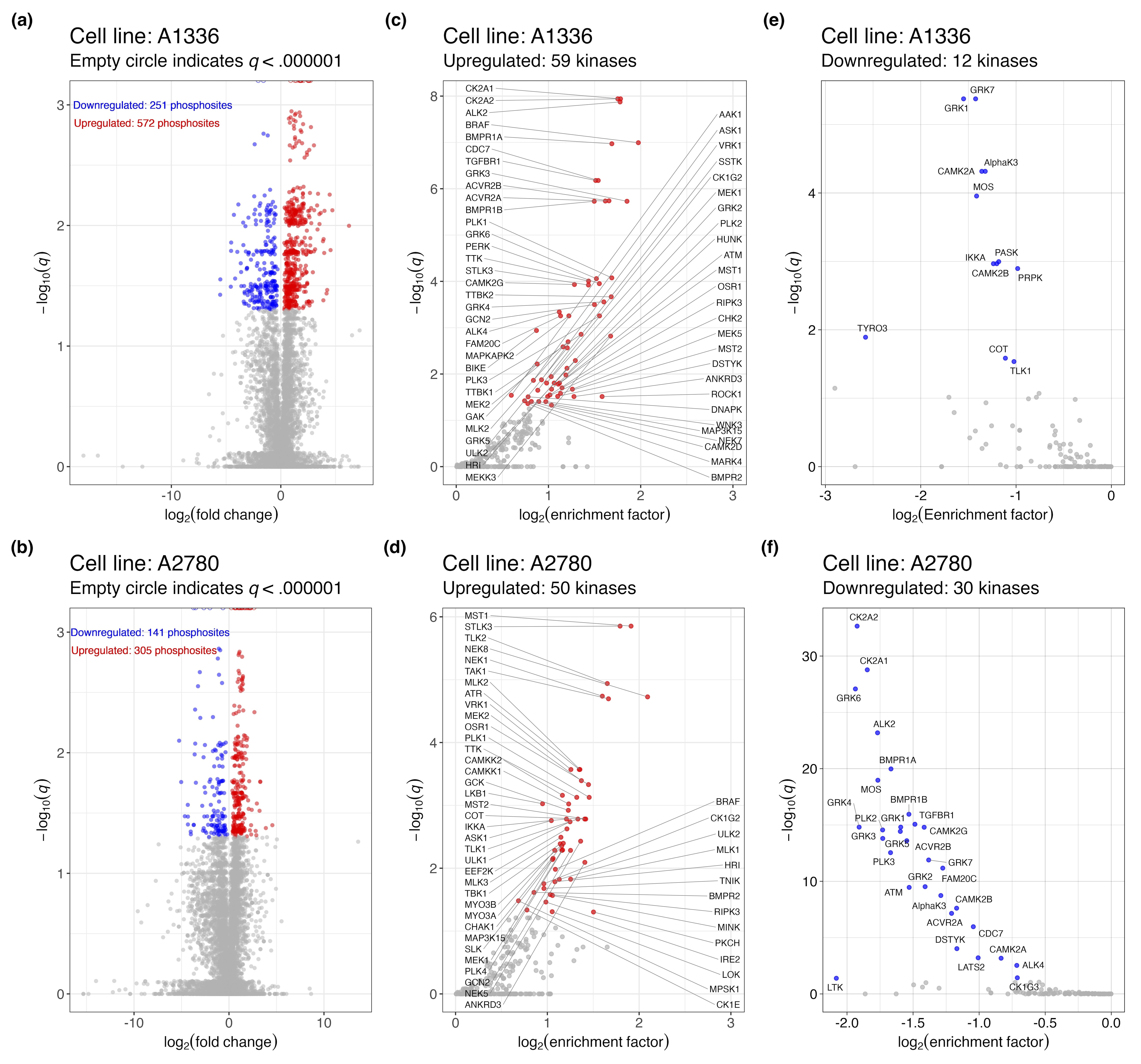

Supplement: Supplementary file 4 — Supporting information [file CTM2-15-e70078-s001.jpg]

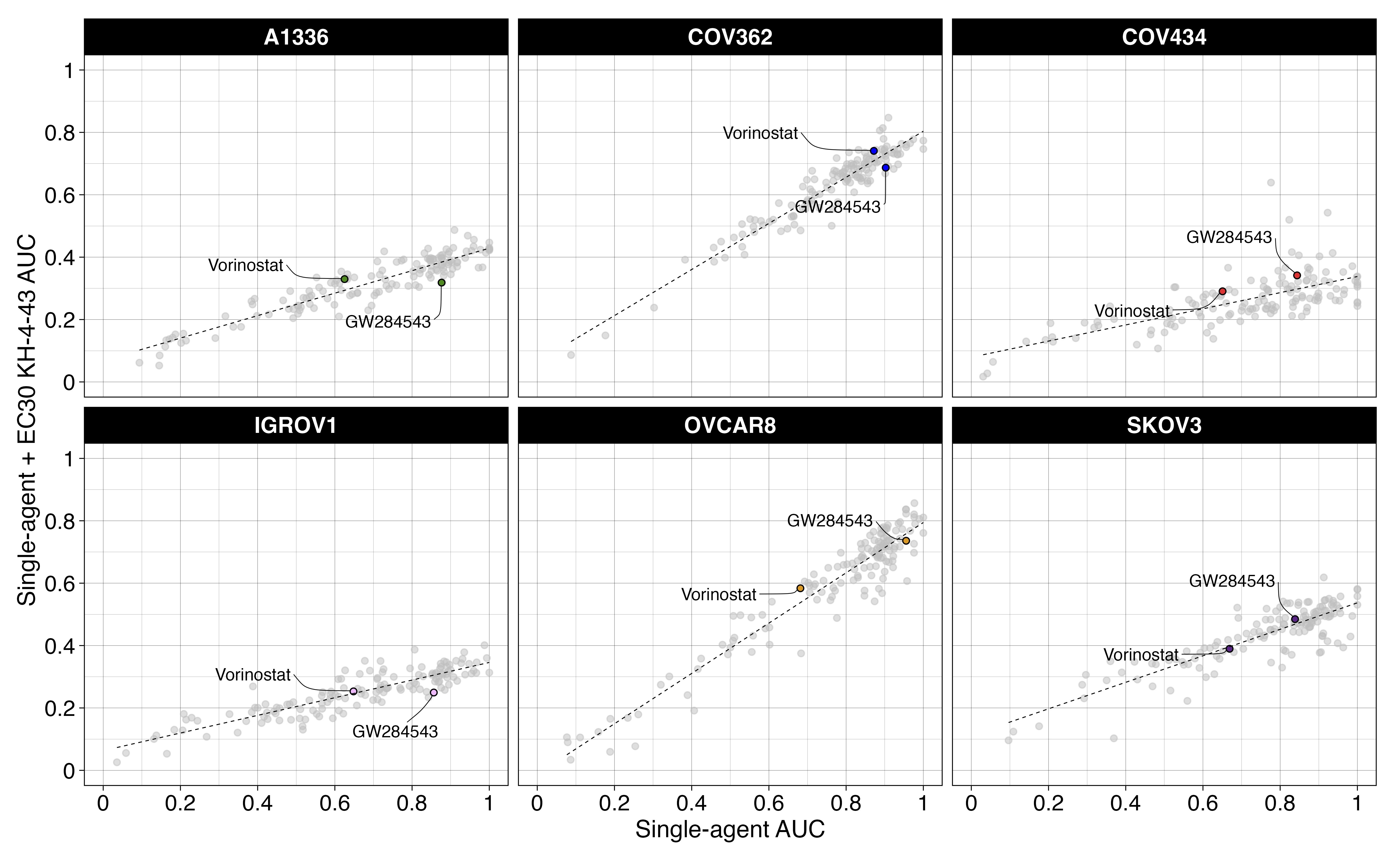

Supplement: Supplementary file 5 — Supporting information [file CTM2-15-e70078-s014.jpg]

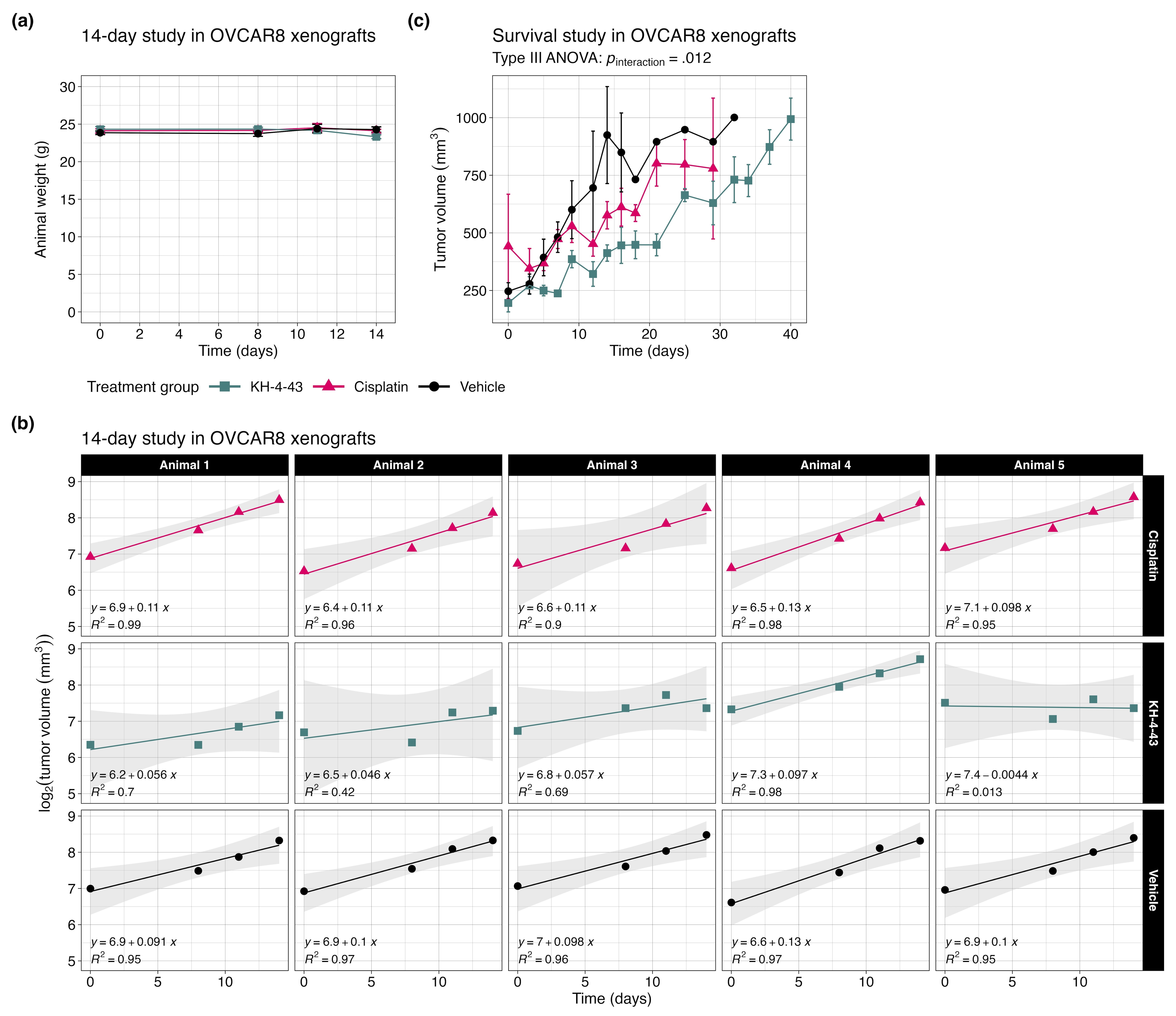

Supplement: Supplementary file 6 — Supporting information [file CTM2-15-e70078-s010.jpg]

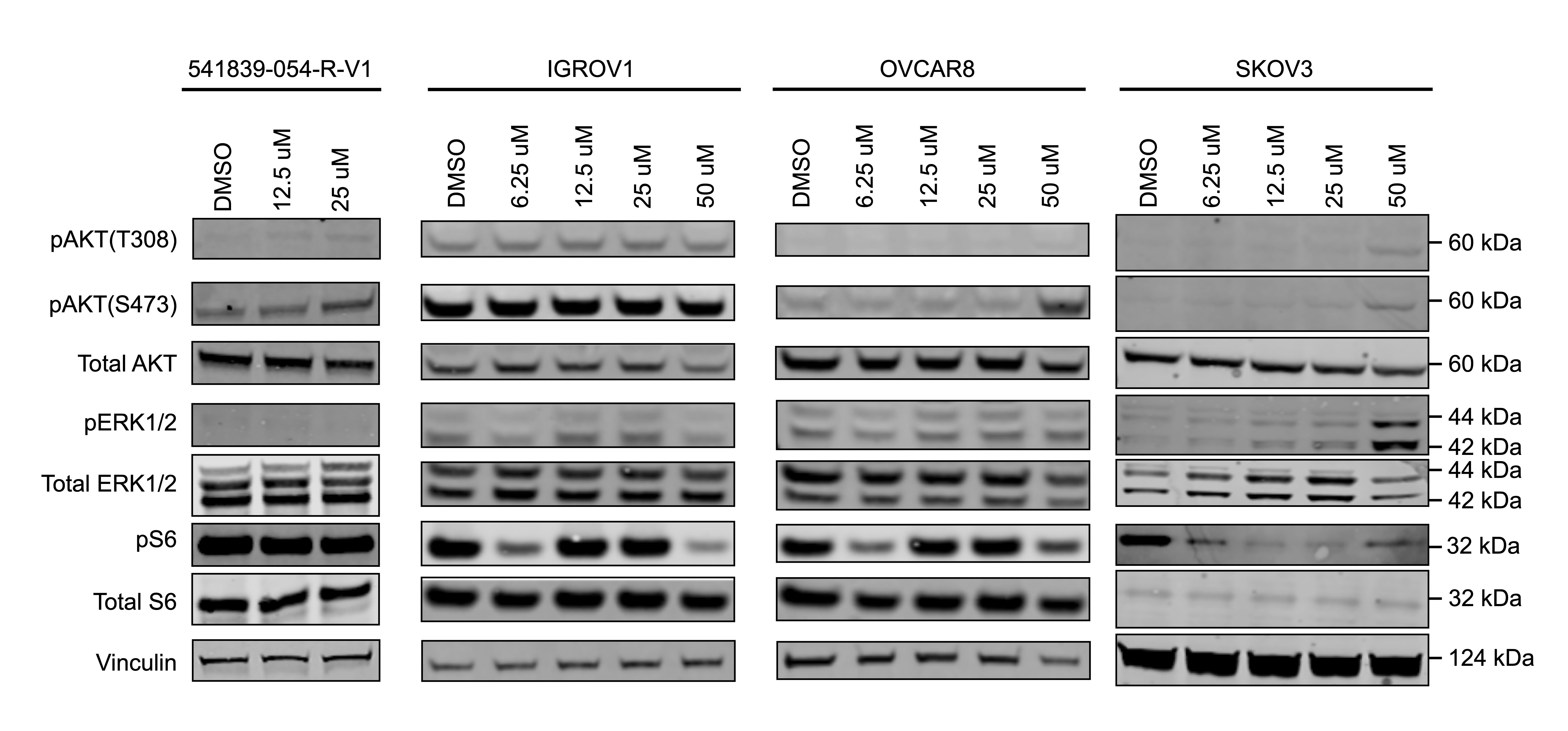

Supplement: Supplementary file 7 — Supporting information [file CTM2-15-e70078-s004.jpg]

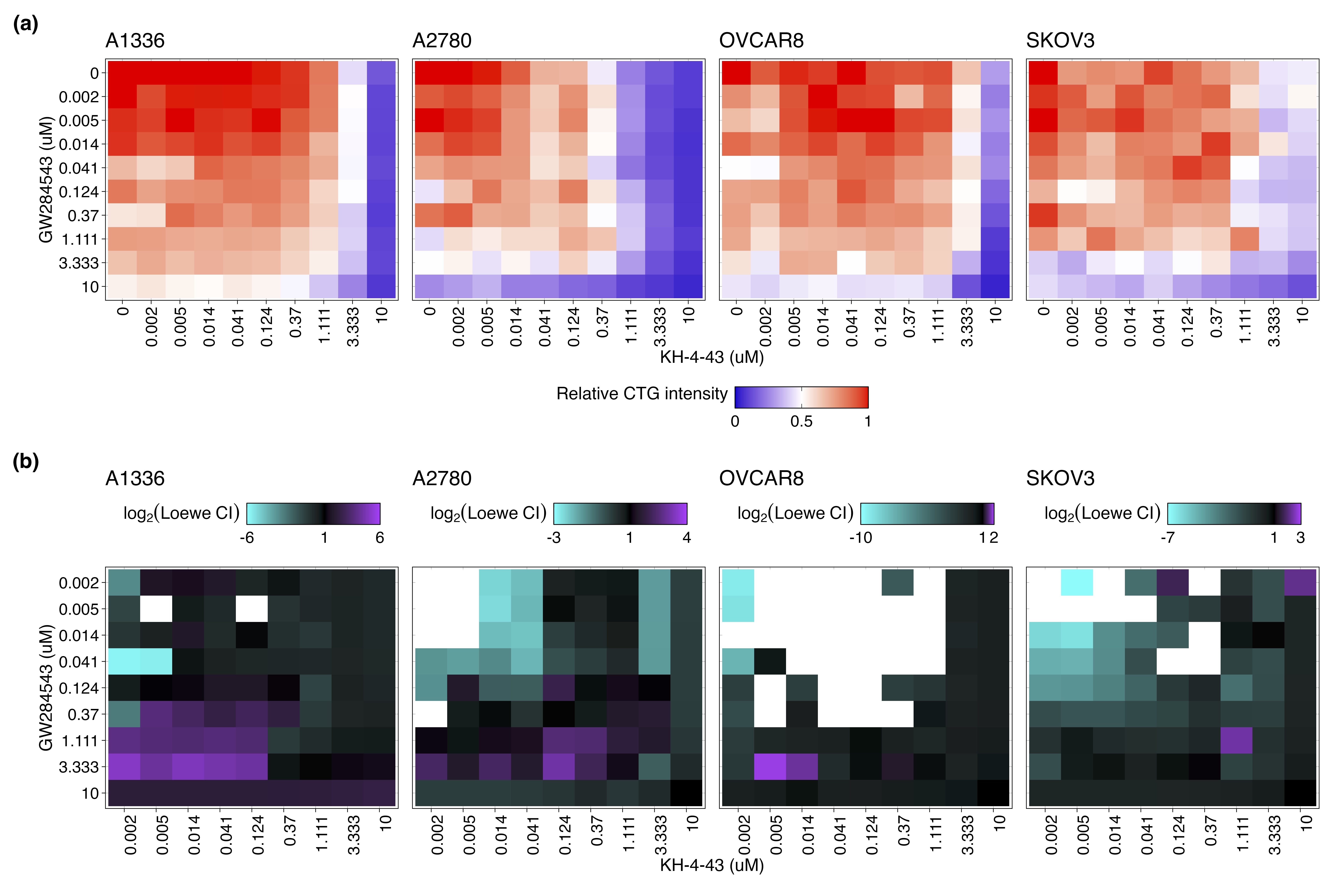

Supplement: Supplementary file 8 — Supporting information [file CTM2-15-e70078-s007.jpg]

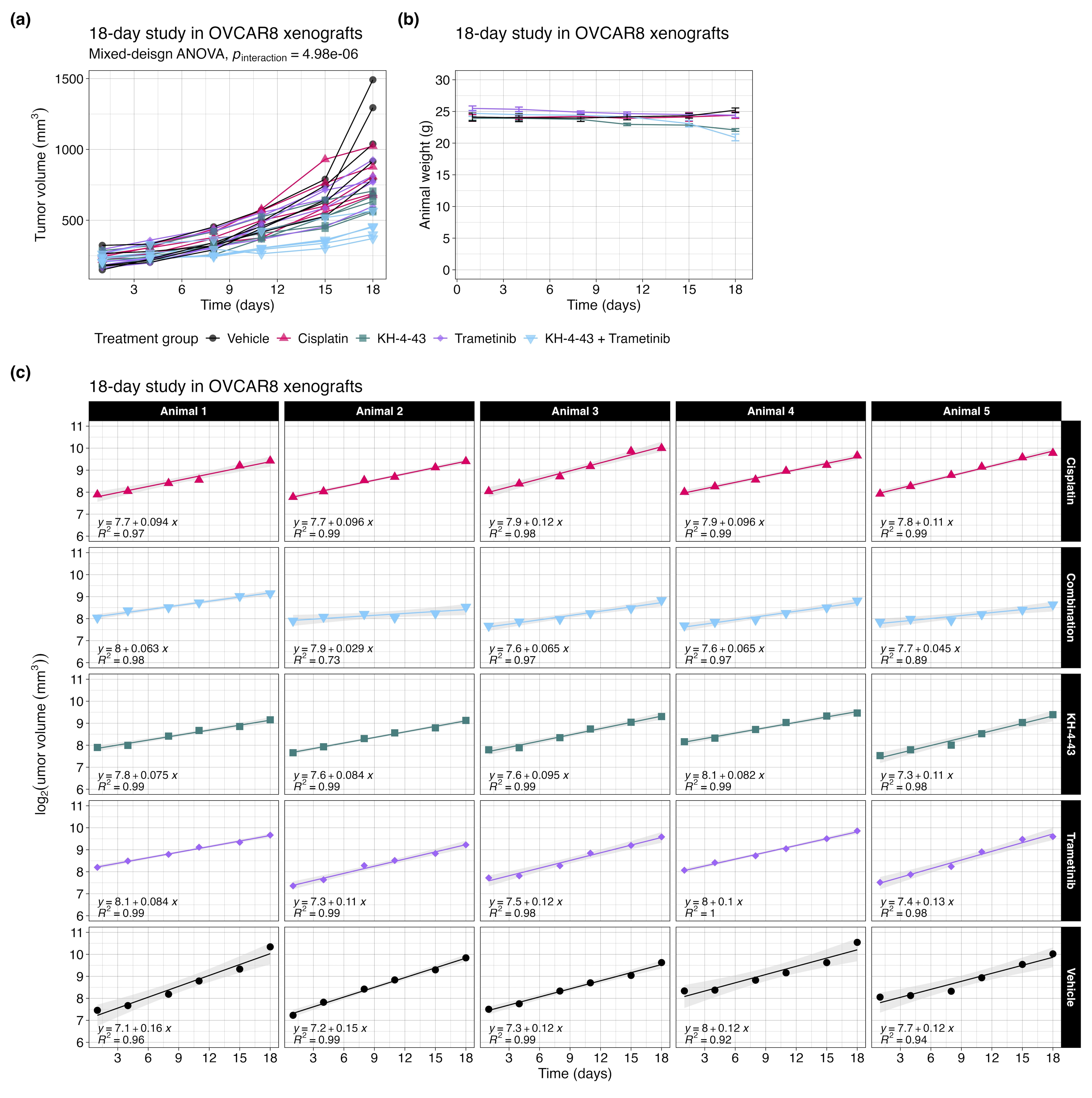

Supplement: Supplementary file 9 — Supporting information [file CTM2-15-e70078-s008.jpg]

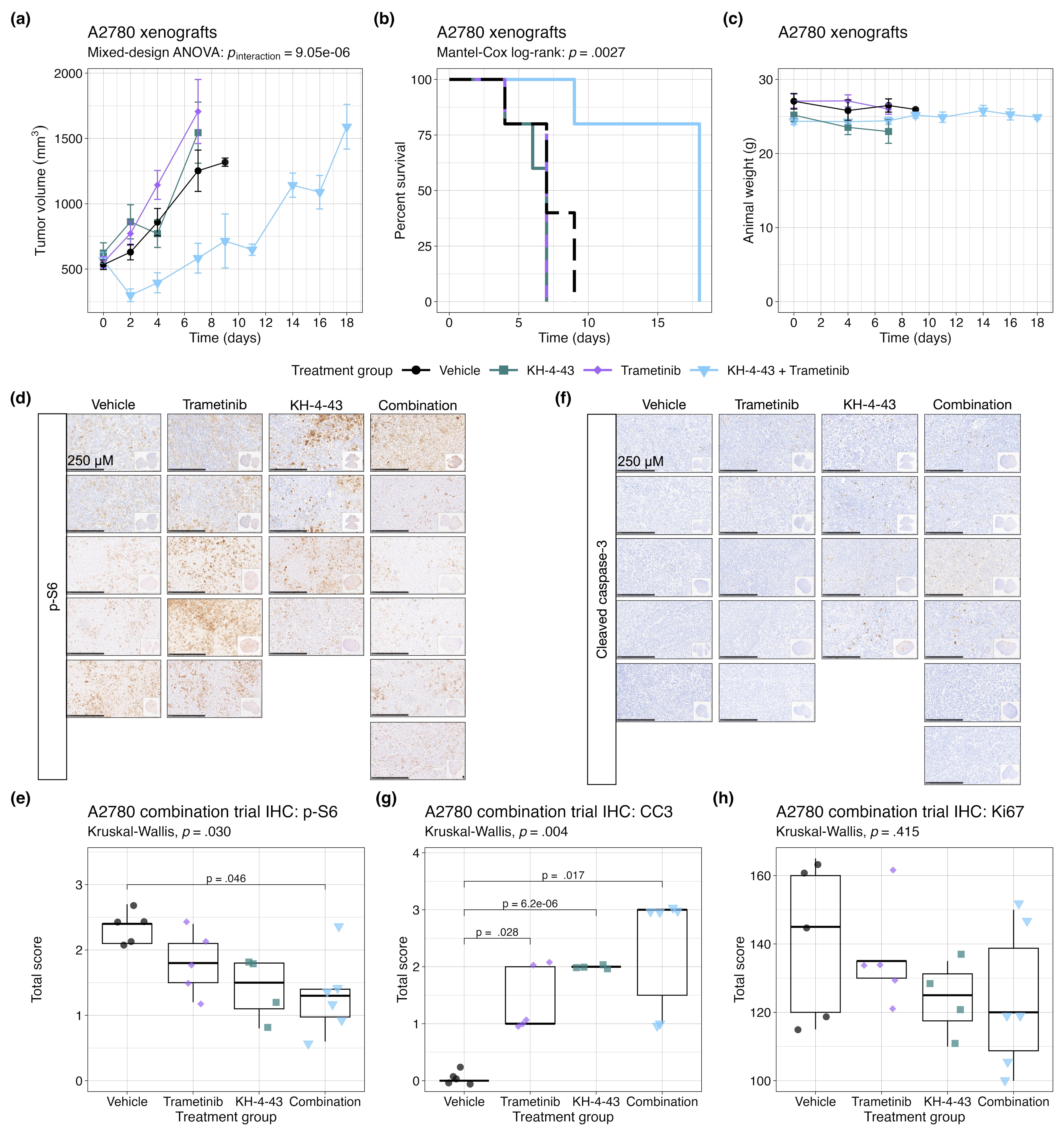

Supplement: Supplementary file 10 — Supporting information [file CTM2-15-e70078-s009.jpg]
