# Supplementary material for: Functional genomics pipeline identifies CRL4 inhibition for the treatment of ovarian cancer
Source: Clin Transl Med. 2025 Jan 24;15(2):e70078. doi: 10.1002/ctm2.70078 (PMC11761363; doi:10.1002/ctm2.70078)
Supplement: Supplementary file 14 — Supporting information [file CTM2-15-e70078-s005.docx]

**Supplementary Table S1. Lineage designations of cancer models used.**
Included are all cell lines and organoids screened in the Functional Genomics Pipeline and their lineages and primary sources (either purchased directly from the American Type Culture Collection or kindly provided by the indicated investigator at the Icahn School of Medicine at Mount Sinai).

| **Line** | **Primary Lineage** | **Source** |
| --- | --- | --- |
| MDA-MB-231 | Breast | American Type Culture Collection |
| MDA-MB-453 | Breast | American Type Culture Collection |
| MDA-MB-468 | Breast | American Type Culture Collection |
| T47D | Breast | American Type Culture Collection |
| A549 | Lung | American Type Culture Collection |
| NCCIT | Testis | American Type Culture Collection |
| TERA-2 | Testis | American Type Culture Collection |
| 92.1 | Uveal Melanoma | Anne Bowcock, PhD |
| Mel202 | Uveal Melanoma | Anne Bowcock, PhD |
| Mel270 | Uveal Melanoma | Anne Bowcock, PhD |
| Mel290 | Uveal Melanoma | Anne Bowcock, PhD |
| MP46 | Uveal Melanoma | Anne Bowcock, PhD |
| MP65 | Uveal Melanoma | Anne Bowcock, PhD |
| H1435 | Lung | Fred Hirsch, MD, PhD |
| H1563 | Lung | Fred Hirsch, MD, PhD |
| H1573 | Lung | Fred Hirsch, MD, PhD |
| H1581 | Lung | Fred Hirsch, MD, PhD |
| H1650 | Lung | Fred Hirsch, MD, PhD |
| H1703 | Lung | Fred Hirsch, MD, PhD |
| H1792 | Lung | Fred Hirsch, MD, PhD |
| H196 | Lung | Fred Hirsch, MD, PhD |
| H1993 | Lung | Fred Hirsch, MD, PhD |
| H2030 | Lung | Fred Hirsch, MD, PhD |
| H2228 | Lung | Fred Hirsch, MD, PhD |
| H292 | Lung | Fred Hirsch, MD, PhD |
| H520 | Lung | Fred Hirsch, MD, PhD |
| H522 | Lung | Fred Hirsch, MD, PhD |
| H596 | Lung | Fred Hirsch, MD, PhD |
| H647 | Lung | Fred Hirsch, MD, PhD |
| HCC15 | Lung | Fred Hirsch, MD, PhD |
| HCC44 | Lung | Fred Hirsch, MD, PhD |
| BT549 | Breast | Jian Jin, PhD |
| A382 | Astrocytoma | Stuart Aaronson, MD^133^ |
| A253 | Epidermoid | Stuart Aaronson, MD^133^ |
| A388 | Epidermoid | Stuart Aaronson, MD^133^ |
| A431 | Epidermoid | Stuart Aaronson, MD^133^ |
| A172 | Glioblastoma | Stuart Aaronson, MD^133^ |
| A498 | Kidney | Stuart Aaronson, MD^133^ |
| A704 | Kidney | Stuart Aaronson, MD^133^ |
| A101D | Melanoma | Stuart Aaronson, MD^133^ |
| A375 | Melanoma | Stuart Aaronson, MD^133^ |
| A875 | Melanoma | Stuart Aaronson, MD^133^ |
| A204 | Rhabdomyosarcoma | Stuart Aaronson, MD^133^ |
| A673 | Rhabdomyosarcoma | Stuart Aaronson, MD^138^ |

| **Ovarian epithelial cancer (OC) cell lines** | | |
| --- | --- | --- |
| **Line** | **OC Subtype** | **Source** |
| SKOV3 | Serous | Bin Zhang, PhD |
| COV362 | High-grade serous | Bin Zhang, PhD |
| COV434 | Small cell carcinoma | Bin Zhang, PhD |
| IGROV1 | Endometrioid | Bin Zhang, PhD |
| OVCAR8 | Low-grade serous^134^ | Bin Zhang, PhD |
| A2780 | Endometrioid | Bin Zhang, PhD |
| A1336 | Unknown | Stuart Aaronson, MD (unpublished) |
| A2780 (parental) | Endometrioid | MilliporeSigma (cat. #93112519) |
| A2780cis | Endometrioid | MilliporeSigma (cat. #93112517) |

| **OC organoid lines** | | |
| --- | --- | --- |
| **Line** | **OC Subtype** | **Source** |
| 541839-054-R-V1 | Mucinous adenocarcinoma | National Cancer Institute Patient-Derived Models Repository |

**Supplementary Table S2. Oncogenic alterations in the ovarian cancer cell lines used in this study.**

Mutation data for the seven commercially available cell lines were referenced from the Precision Oncology Knowledgebase (OncoKB) (v4.7) ^135^ annotations curated by DepMap that are available via the DepMap Data Portal^136^. Note that no OncoKB-annotated alterations were recorded in COV434 nor OVSAHO cells, and because A1336 is not a published cell line, alterations were referenced from Oncomine Comprehensive Assay sequencing data. Note that none of the mutations in A1336 were annotated but their oncogenicity and function were inferred from OncoKB. GOF: Gain-of-function, LOF: Loss-of-function.

| Cell line | Gene | Full name | Alteration | Oncogenicity | Function |
| --- | --- | --- | --- | --- | --- |
| A2780 | *RRAS2* | Ras-related 2 | Q72L | Oncogenic | GOF |
|  | *SMARCA4* | SWI/SNF-related, matrix-associated, actin-dependent regulator of chromatin, subfamily A, member 4 | T910M | Likely oncogenic | LOF |
|  | *PIK3CA* | Phosphatidylinositol-4,5-bisphosphate 3-kinase catalytic subunit alpha | E365K | Likely oncogenic | GOF |
|  | *MED12* | Mediator complex subunit 12 | D23Y | Oncogenic | Likely LOF |
| COV362 | *TP53* | Tumor protein 53 | Y220C | Oncogenic | LOF |
|  | *LZTR1* | Leucine zipper-like post translational regulator 1 | R466Q | Oncogenic | Likely LOF |
| SKOV3 | *EP300* | E1A binding protein P300 | Y1414C | Likely oncogenic | LOF |
|  | *TP53* | Tumor protein 53 | H179R^137^ | Oncogenic | LOF |
|  | *PIK3CA* | Phosphatidylinositol-4,5-bisphosphate 3-kinase catalytic subunit alpha | H1047R | Oncogenic | GOF |
| OVCAR8 | *ERBB2* | Erythroblastic oncogene B receptor tyrosine kinase 2 (also known as HER2) | G776V | Oncogenic | GOF |
| OVCAR3 | *TP53* | Tumor protein 53 | R248Q | Likely oncogenic | Likely LOF |
| IGROV1 | *PTEN* | Phosphatase and tensin homolog | Y155C | Oncogenic | LOF |
| IGROV1 | *PTPN11* | Protein tyrosine phosphatase non-receptor type 11 | Y62H | Likely oncogenic | Likely LOF |
|  | *TP53* | Tumor protein 53 | Y126C | Likely oncogenic | Likely LOF |
|  | *PIK3CA* | Phosphatidylinositol-4,5-bisphosphate 3-kinase catalytic subunit alpha | R38C | Likely oncogenic | Likely GOF |
| A1336 | *ARID1A* | AT-rich interaction domain 1A | S634* | Likely oncogenic | Likely LOF |
|  | *NF1* | Nuclear factor 1 | E977* | Likely oncogenic | Likely LOF |
|  | *PTEN* | Phosphatase and tensin homolog | C211* | Likely oncogenic | Likely LOF |

**Supplementary Table S3.** **Human KEGG pathways associated with signal transduction.**

Human KEGG pathways that were used to generate a comprehensive list of MAPK-related signaling genes.

| **Pathway identifier** | **Pathway name** | **Number of genes** |
| --- | --- | --- |
| hsa04010 | MAPK_signaling_pathway | 300 |
| hsa04012 | ErbB_signaling_pathway | 86 |
| hsa04014 | Ras_signaling_pathway | 238 |
| hsa04015 | Rap1_signaling_pathway | 212 |
| hsa04310 | Wnt_signaling_pathway | 174 |
| hsa04330 | Notch_signaling_pathway | 62 |
| hsa04340 | Hedgehog_signaling_pathway | 56 |
| hsa04350 | TGF-beta_signaling_pathway | 108 |
| hsa04390 | Hippo_signaling_pathway | 157 |
| hsa04370 | VEGF_signaling_pathway | 60 |
| hsa04371 | Apelin_signaling_pathway | 140 |
| hsa04630 | JAK-STAT_signaling_pathway | 168 |
| hsa04064 | NF-kappa_B_signaling_pathway | 105 |
| hsa04668 | TNF_signaling_pathway | 119 |
| hsa04066 | HIF-1_signaling_pathway | 109 |
| hsa04068 | FoxO_signaling_pathway | 133 |
| hsa04020 | Calcium_signaling_pathway | 254 |
| hsa04070 | Phosphatidylinositol_signaling_system | 98 |
| hsa04072 | Phospholipase_D_signaling_pathway | 149 |
| hsa04071 | Sphingolipid_signaling_pathway | 122 |
| hsa04024 | cAMP_signaling_pathway | 226 |
| hsa04022 | cGMP-PKG_signaling_pathway | 166 |
| hsa04151 | PI3K-Akt_signaling_pathway | 362 |
| hsa04152 | AMPK_signaling_pathway | 122 |
| hsa04150 | mTOR_signaling_pathway | 158 |

Table S4. Summary of the Kinase Library differential phosphosite enrichment analysis in KH-4-43-treated A1336 and A2780 cells.

Background sites are determined by |log_2_(Fold Change)| < 1.

| Cell Line | Input | Serine/Threonine phosphorylation sites | | | Tyrosine phosphorylation sites | | |
| --- | --- | --- | --- | --- | --- | --- | --- |
|  |  | Upregulated | Downregulated | Background | Upregulated | Downregulated | Background |
| A1336 | 5,754 | 420 | 509 | 4,651 | 26 | 27 | 121 |
| A2780 | 7,478 | 393 | 802 | 6,064 | 15 | 39 | 165 |

# REFERENCES

133. Giard, D. J. *et al.* In Vitro Cultivation of Human Tumors: Establishment of Cell Lines Derived From a Series of Solid Tumors2. *JNCI: Journal of the National Cancer Institute* **51**, 1417–1423 (1973).

134. McCabe, A., Zaheed, O., McDade, S. S. & Dean, K. Investigating the suitability of in vitro cell lines as models for the major subtypes of epithelial ovarian cancer. *Front. Cell Dev. Biol.* **11**, 1104514 (2023).

135. Chakravarty, D. *et al.* OncoKB: A Precision Oncology Knowledge Base. *JCO Precision Oncology* 1–16 (2017) doi:10.1200/PO.17.00011.

136. Tsherniak, A. *et al.* Defining a Cancer Dependency Map. *Cell* **170**, 564-576.e16 (2017).

137. Mullany, L. K. *et al.* Specific TP53 Mutants Overrepresented in Ovarian Cancer Impact CNV, TP53 Activity, Responses to Nutlin-3a, and Cell Survival. *Neoplasia* **17**, 789–803 (2015).
